# Autonomic Integration in Nested Protocell Communities

**DOI:** 10.1021/jacs.3c02816

**Published:** 2023-06-27

**Authors:** Zhuping Yin, Ning Gao, Can Xu, Mei Li, Stephen Mann

**Affiliations:** † Centre for Protolife Research, School of Chemistry, University of Bristol, Bristol BS8 1TS, UK; ‡ Max Planck-Bristol Centre for Minimal Biology, School of Chemistry, University of Bristol, Bristol BS8 1TS, UK; § School of Materials Science and Engineering, Shanghai Jiao Tong University, Shanghai 200240, P. R. China; ∥ Zhangjiang Institute for Advanced Study (ZIAS), Shanghai Jiao Tong University, 429 Zhangheng Road, Shanghai 201203, P. R. China

## Abstract

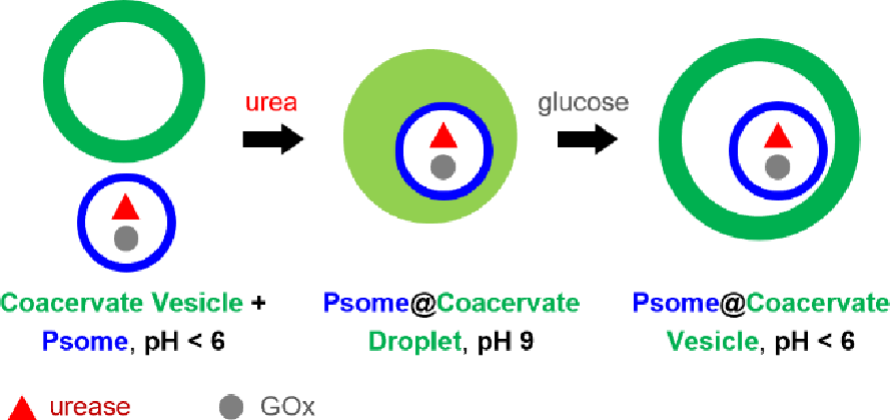

The self-driven organization of model protocells into higher-order nested cytomimetic systems with coordinated structural and functional relationships offers a step toward the autonomic implementation of artificial multicellularity. Here, we describe an endosymbiotic-like pathway in which proteinosomes are captured within membranized alginate/silk fibroin coacervate vesicles by guest-mediated reconfiguration of the host protocells. We demonstrate that interchange of coacervate vesicle and droplet morphologies through proteinosome-mediated urease/glucose oxidase activity produces discrete nested communities capable of integrated catalytic activity and selective disintegration. The self-driving capacity is modulated by an internalized fuel-driven process using starch hydrolases sequestered within the host coacervate phase, and structural stabilization of the integrated protocell populations can be achieved by on-site enzyme-mediated matrix reinforcement involving dipeptide supramolecular assembly or tyramine–alginate covalent cross-linking. Our work highlights a semi-autonomous mechanism for constructing symbiotic cell-like nested communities and provides opportunities for the development of reconfigurable cytomimetic materials with structural, functional, and organizational complexity.

## Introduction

Synthetic protobiology is concerned with the construction, reconstitution, and functionalization of individual cell-like entities,^1−8^ development of living cell/synthetic cell constructs (cellular bionics) and signaling networks,^9−16^ and implementation of protocell dynamics and chemical communication in populations of artificial cells.^17−21^ Studies of dispersed protocell communities explore contact-dependent and through-space chemical interactions as steps toward the development of higher-order cytomimetic behaviors such as division and growth,^22^ prototissue assembly,^23−27^ signal processing,^6,28−32^ self-sorting,^33^ and DNA-based computation.^34,35^ Nested communities of synthetic cells can be prepared by sequential processing or microfluidic techniques to produce vesicle-in-vesicle,^36^ proteinosome-in-proteinosome,^37^ polymersome-in-polymersome,^38^ polymersome-in-polymer capsule,^39^ coacervate-in-coacervate droplet,^40^ and living cell-in-vesicle^41,42^ arrangements. Alternatively, properties such as wetting and interfacial tension can be utilized for the development of physically interactive artificial cell communities that exhibit behaviors such as artificial phagocytosis,^43^ parasitism,^44^ and predation^45−48^ to produce nested protocells via mechanisms of engulfment, processing, and functional integration (artificial endosymbiosis). In general, these mechanisms involve the use of molecularly crowded coacervate droplets that display a membrane-less interfacial barrier, which facilitates the capture of guest protocells by contact-dependent attractive interactions with the exposed coacervate matrix. As the hybrid constructs are inherently unstable due to coacervate droplet–droplet coalescence, recent studies have used membranized coacervates^49−52^ with triggerable membrane dynamics to control the selective uptake and integration of guest protocells in response to light irradiation or enzyme-mediated changes in pH.^53^ Because such approaches are compromised by irreversible depletion of the membrane building blocks and concomitant structural instability, in this paper, we develop a self-driving process of reversible coacervate membranization, which offers increased robustness and functional adaptation in cell-like nested communities and provides an autonomous mechanism for the construction of artificial multicellularity via the chemical programming of interactive protocell behavior.

Membranized coacervate-based protocells can be spontaneously generated through coacervate droplet-to-vesicle reconfigurations by employing auxiliary surface reconstruction agents or triggering endogenous structural transitions (self-membranization) under nonstoichiometric conditions.^49^ For example, self-membranization of silk-based coacervate droplets produces osmotically inflated semi-permeable coacervate vesicles and modulates the trafficking of low-molecular weight solutes.^54^ In this paper, we implement the self-driven capture of proteinosomes within reconfigurable alginate/silk fibroin coacervate vesicles to establish the autonomic integration of the different protocell populations. We show that the capture of single or binary populations of proteinosomes^55,56^ by preformed single-chambered coacervate vesicles can be achieved via the reversible interchange of the coacervate vesicle and droplet morphologies through programmed urease/glucose oxidase (GOx) activity specifically within the proteinosomes. Moreover, sequestration of starch hydrolases within the host coacervate phase modulates the self-driving capacity via an internalized fuel-driven process, thereby establishing primitive levels of host/guest communication and cooperativity in the endosymbiotic-like pathway. We demonstrate that capture of the proteinosomes into multi-chambered coacervate vesicles with increased structural robustness can be achieved by guest- or host-mediated hydrogelation of the coacervate phase using on-site enzyme-mediated dipeptide supramolecular assembly or covalent cross-linking of tyramine–alginate, respectively. The integrated protocell populations are exploited as catalytic host–guest constructs and undergo selective membrane disassembly when challenged by a reducing agent in the external environment. Overall, our work highlights a semi-autonomous mechanism for constructing symbiotic cell-like nested communities capable of self-induced matrix reinforcement, endogenously coupled catalytic reactivity, and selective membrane disintegration and provides opportunities for the development of reconfigurable cytomimetic materials with structural, functional, and organizational complexity.

## Results and Discussion

### Self-Driven Capture of Proteinosomes in Reconfigurable Coacervate Vesicles

Cationized silk fibroin (CSF; zeta potential +25 mV; disulfide-cross-linked heavy chain, *M*_w_ ≈ 390 kDa; light chain, *M*_w_ ≈ 26 kDa) was prepared as previously reported (Figure S1)^54^ and mixed with negatively charged alginate (*M*_w_ = 140–160 kDa) to induce coacervation by associative liquid–liquid phase separation. Depending on the conditions, near-to-neutral membrane-less coacervate droplets, multi-compartmentalized coacervate vesicles, and positively or negatively charged coacervate vesicles were produced and reversibly interchanged by alterations in the alginate [COOH]:CSF [NH_2_] charge ratio (Figure S2). The coacervate vesicles consisted of single or multiple osmotically expanded water-filled vacuoles that were caged within a continuous and compressed coacervate phase that served as a barrier to macromolecular uptake (Figure S3). Significantly, the positively charged coacervate vesicles were formed via a self-membranization process arising from the segregation of excess amphiphilic CSF chains at the coacervate droplet/water interface under non-stoichiometric (charge-mismatched) conditions.^54^ Consequently, the coacervate vesicles were reversibly reconfigured into membrane-free alginate/CSF coacervate droplets by increasing the degree of alginate deprotonation by increasing the pH above 8, or adding additional alginate to give alginate: CSF charge ratios above 2:1 (Figures S2, S4, and S5). Alternatively, populations of silk-based coacervate droplets were transformed into suspensions of positively charged coacervate vesicles by decreasing the pH below 6 or adding CSF to change the charge ratio to less than 1:1 (Figures S2, S4, and S5).

**Figure 1:**
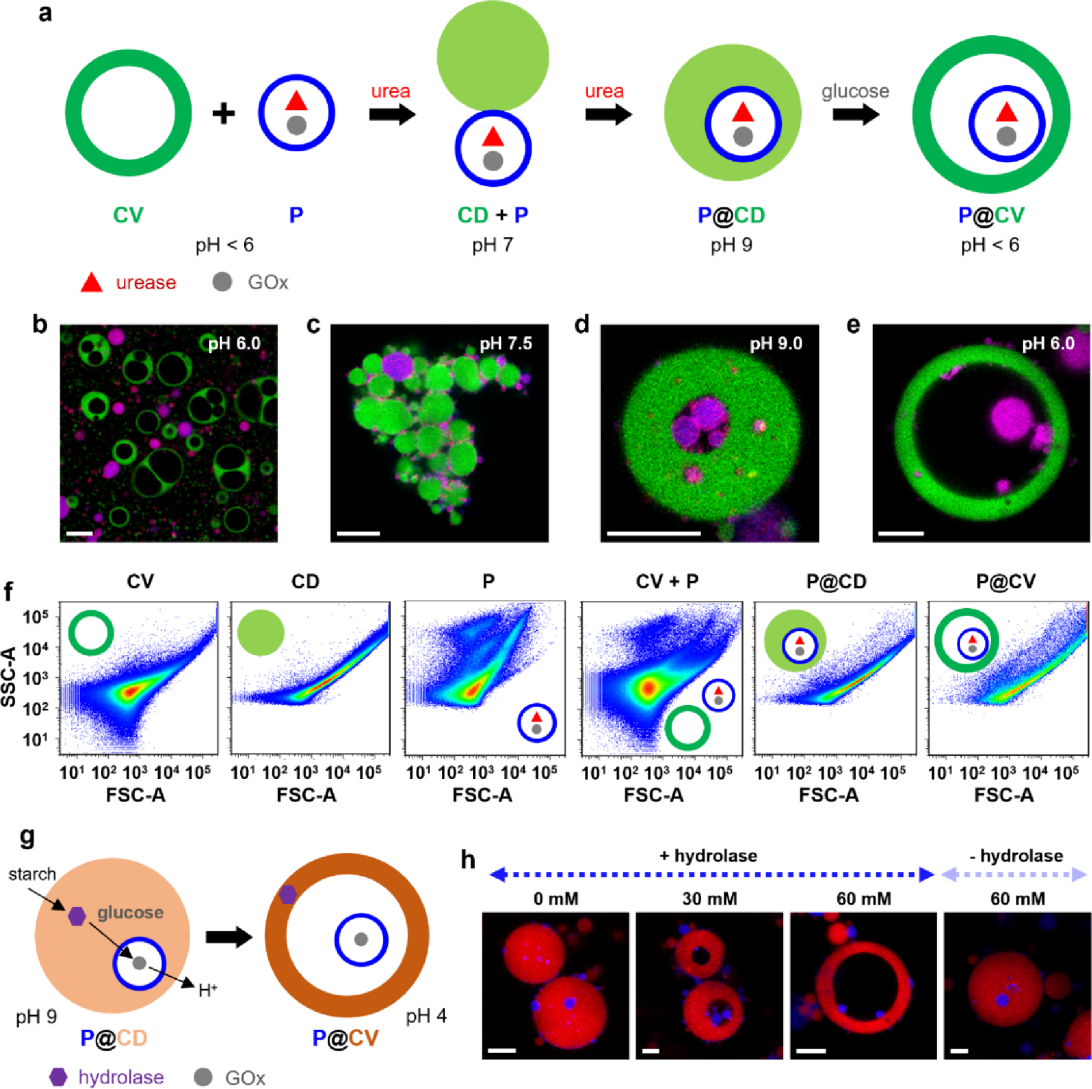
Self-driven capture of proteinosomes in reconfigurable coacervate vesicles. (a) Urease (red triangles)/GOx (gray dots)-containing proteinosomes (P, blue rings) are incorporated into alginate/CSF coacervate vesicles (CV, dark green rings) by endogenously controlled enzyme-mediated reconfiguration via a two-step pH-oscillation process involving membrane-less coacervate droplets (CD, green circles) intermediates. A CV and P non-interacting binary population at pH 4 is transformed into an interacting CD + P community at pH 7 by urea/urease activity, and results in spontaneous proteinosome capture and formation of proteinosome-in-coacervate droplets (P@CD) at pH 9. P@CD is reconfigured into proteinosome-in-coacervate vesicles (P@CV) at pH 4 by GOx/glucose-mediated self-membranization. (b–e) LSCM images showing a non-interacting binary population of coacervate vesicles (FITC-CSF, green fluorescence) and proteinosomes (Dylight 405, blue fluorescence) with encapsulated GOx, urease, and RITC-gelatin (red fluorescence) at pH 6 (b), interactive binary population of proteinosomes (purple) and coacervate droplets (green) at pH 7.5 (c), a single coacervate droplet with captured proteinosomes at pH 9 (d), and an individual proteinosome-in-coacervate vesicle at pH 6 (e); scale bars, 20 μm. (f) FACS 2D dot plots of forward light scattered area (FSC-A) versus side light scattered area (SSC-A) for single populations of CV, CD and P, a non-interacting CV + P binary population, and populations of P@CD and P@CV. Total number of particles recorded in each experiment: *n* = 20,000. Variance values; σ^2^ = 0.24, 0.65, and 1.23 for CV, P@CD, and P@CV, respectively. (g) Fuel-driven reconfiguration of P@CD into P@CV by enzyme-mediated cascade processing. P@CD is prepared at pH 9 with sequestered starch hydrolases (purple hexagons) and captured GOx-containing proteinosomes (gray dots in blue rings). Addition of soluble starch induces the endogenous production of glucose, GOx-mediated acidification, and membranization. (h) LSCM images of hydrolase/GOx-containing P@CD before (left) and 10.5 h after addition of starch at concentrations of 30 or 60 mM showing the formation of P@CV. No reconfiguration is observed when starch (60 mM) is added in the absence of the starch hydrolases (right image). Amylase (40 IU/mL); amyloglucosidase (20 IU/mL); proteinosomes (Dylight 405, blue fluorescence); alginate/RITC-CSF coacervate (red fluorescence). Scale bars, 20 μm.

Given the facile and reversible nature of self-membranization, we developed a self-driving two-step enzyme-mediated pathway for the chemically induced capture of guest protocells (proteinosomes; Figure S6) into the positively charged alginate/CSF coacervate vesicles (Figure 1a). The membrane-bounded proteinosomes^55,56^ were cross-linked with *O*,*O*′-bis[2-(*N*-succinimidyl-succinylamino)ethyl]polyethylene glycol (PEG-NHS; *M*_w_ = 2000) and contained co-encapsulated urease and glucose oxidase (GOx) as endogenous agents for increasing (production of NH_3_) or decreasing (production of gluconic acid) the pH, respectively, along with encapsulated aqueous gelatin to stabilize the proteinosomes against changes in osmotic pressure. Significantly, in the absence of enzyme activity, binary populations of coacervate vesicles (mean size, 11.5 μm; range, 1.5–30 μm) and proteinosomes (mean diameter, 5 μm; range, 3–15 μm) were non-interacting over 24 h (Figure 1b and Figure S7). The absence of fusion and wetting of the different populations indicated that the coacervate vesicle membrane was an effective physical barrier to proteinosome uptake. In contrast, the addition of urea to the mixed populations gave rise within 2 h to an increase in pH from 4 to 8.3 due to urease activity within the proteinosomes (Figure S8). Consequently, the coacervate vesicles were transformed into membrane-less coacervate droplets that at pH 7–8 began to coalesce, adventitiously entrapping some of the proteinosomes (Figure 1c and Figure S9). As the pH increased to 9, spontaneous contact and rearrangement between the coacervate droplets and proteinosomes resulted in well-defined proteinosome-in-coacervate droplets (Figure 1d and Figure S9). To re-establish the coacervate vesicle membrane, glucose was added to re-acidify the medium by endogenous proteinosome-based GOx activity (Figure S8). Decreases in pH to ca. 7.5 resulted in the formation of multiple vacuoles that continued to expand until they coalesced into a single water-filled lumen at pH 6 to produce discrete alginate/CSF coacervate vesicles with the guest proteinosomes expelled from the compressed coacervate phase into the lumen (Figure 1e). A thin highly compressed coacervate membrane domain was observed at pH 4 along with internally trapped proteinosomes (Figure S10). The proteinosome capture efficiency was ca. 30% for binary populations prepared at an initial proteinosome:coacervate vesicle number ratio of 1:2. Increasing the relative number of coacervate vesicles increased the capture efficiency of the two-step pathway to values of 48 and 62% at proteinosome:coacervate vesicle ratios of 1:3 and 1:10, respectively. Non-captured proteinosomes were often associated with the surface of the coacervate droplets/vesicles.

Fluorescence-activated cell sorting (FACS) analysis of single populations of positively charged alginate/CSF coacervate vesicles, membrane-less alginate/CSF coacervate droplets, or enzyme-loaded proteinosomes displayed 2D pseudo-color dot plots that were distinguishable due to differences in granularity and particle size (Figure 1f). Mixing suspensions of the coacervate vesicles and urease/GOx/gelatin-containing proteinosomes gave highly dispersed dot plots that were a combination of the two separate populations, confirming the non-interactive nature of these membranized cell-like entities (Figure 1f). In contrast, the addition of urea to generate coacervate droplets with captured proteinosomes resulted in dot plots that were similar to those determined for single populations of the coacervate droplets (Figure 1f), consistent with disassembly of the coacervate vesicles and association of the proteinosomes with the newly formed coacervate droplets. Subsequent addition of glucose to reconfigure the hybrid coacervate droplets into proteinosome-in-coacervate vesicles was accompanied by an increase in the polydispersity (Figure 1f), consistent with the increased heterogeneity observed in mixed populations after the two enzyme-mediated processing steps.

Given the above observations, we sought to modulate the self-driving capacity of the guest proteinosomes by establishing host/guest-dependent membranization in the capture pathway. To achieve this, a fuel-driven enzyme cascade reaction was established across the interface between the captured GOx/gelatin-containing proteinosomes and host coacervate droplet (Figure 1g). Starch hydrolases (amylase and amyloglucosidase) were spontaneously sequestered into the host/guest coacervate droplets (Figure S11) and starch added to the external environment. Uptake of starch into the coacervate droplets (Figure S11) initiated an endogenous enzyme cascade in which the digestion of starch to glucose (Figure S12) within the hydrolase-containing coacervate phase resulted in the diffusive transfer of glucose to the captured proteinosomes. In turn, this resulted in GOx-mediated acidification and reconfiguration into the proteinosome-in-coacervate vesicles (Figure 1h). Increasing the starch concentration resulted in faster rates of coacervate droplet-to-vesicle transformation, while no reconfiguration was observed when starch was added in the absence of the hydrolases, confirming the requirement for host/guest communication and cooperativity.

### Matrix Reinforcement in the Proteinosome-in-Coacervate Vesicles

**Figure 2:**
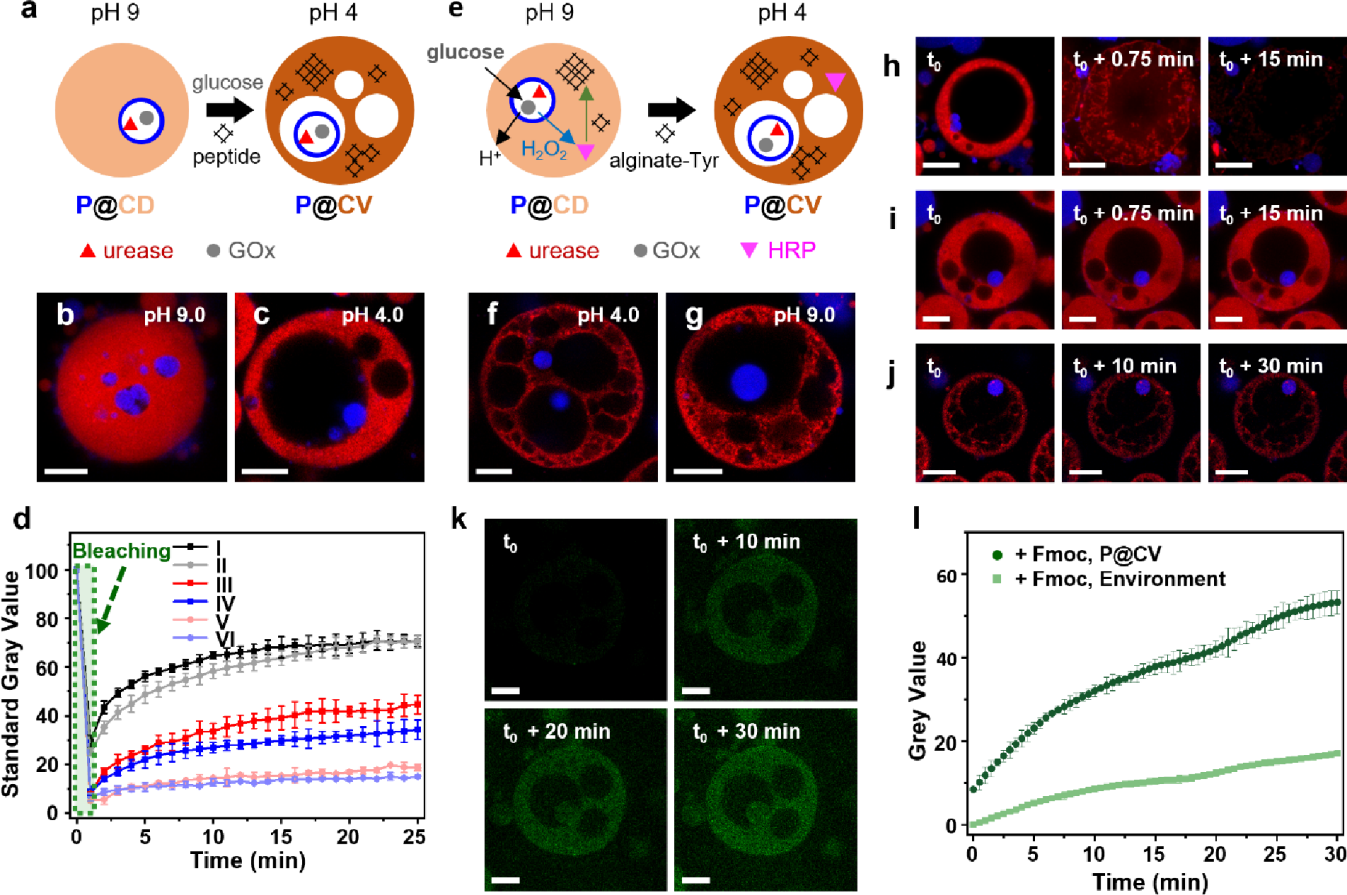
Matrix reinforcement in the proteinosome-in-coacervate vesicles. (a) Guest-mediated peptide assembly mechanism in alginate/CSF scaffolds. A proteinosome-in-coacervate droplet (P@CD) consisting of a membrane-less coacervate phase (light orange circle) and captured urease/GOx-containing proteinosomes (red triangles/gray dots/blue rings) is reconfigured in the presence of glucose and dipeptide Fmoc-AA-OH into a structurally reinforced multi-chambered proteinosome-in-coacervate vesicle (P@CV, dark orange). Membranization and peptide assembly (black symbols) occur simultaneously as the pH is decreased from 9 to 4 by GOx-mediated acidification. (b, c) LSCM images of a single GOx-containing P@CD (b) and P@CV (c) prepared in the presence of Fmoc-AA-OH at pH values of 9 or 4, respectively. The P@CV is structurally reinforced by peptide nanofilaments after the addition of glucose. Fluorescent labels: proteinosomes (Dylight 405, blue fluorescence); coacervate (RITC-CSF, red fluorescence). Scale bars, 20 μm. (d) FRAP profiles (gray values) for P@CD (I, black squares, pH 9, no glucose; II, gray circles, pH 9, no glucose, Fmoc-AA-OH (0.75 mg/mL)); non-reinforced P@CV (III, red squares, pH 4, 20 mM glucose; IV, blue squares, pH 4, 60 mM glucose), and reinforced P@CV (V, orange circles, pH 4, 20 mM glucose, Fmoc-AA-OH (0.75 mg/mL); VI, light purple circles, pH 4, 60 mM glucose, Fmoc-AA-OH (0.75 mg/mL)). The photo-bleaching regime is shaded in green. (e) Host-mediated cross-linking mechanism in alginate-Tyr/CSF scaffolds. Reconfiguration of P@CD at pH 9 into matrix-reinforced multi-chambered P@CV at pH 4 occurs by GOx/HRP-mediated oxidative cross-linking of alginate-Tyr using in situ hydrogen peroxide production after addition of glucose. Increasing the pH back to 9 by urea/urease activity does not reconfigure the reinforced P@CV. Labels as in (a) except for HRP (pink triangles) and alginate-Tyr (black symbols). (f, g) LSCM images of a single P@CV after alginate-Tyr cross-linking recorded at pH 4 (left) and 9 (right). Staining as in (b) and (c). Scale bars, 20 μm. (h–j) Time series of LSCM images showing P@CV at pH 4 with no matrix reinforcement (h), reinforcement with peptide nanofilaments (i), or reinforcement by covalent cross-linking (j) in the presence of sodium chloride. Only the reinforced P@CV are salt-tolerant. Staining as in (b) and (c). Scale bars, 20 μm. (k) Time-dependent LSCM images of a Fmoc-AA-OH-reinforced P@CV containing acid-tolerant lipase before (*t*_0_) and after addition of calcein-AM and sodium chloride. Lipase hydrolase activity is maintained under high ionic strength, producing a calcein (green fluorescence) output. Scale bars are 20 μm. (l) Corresponding changes in calcein fluorescence intensity (gray value) recorded within peptide-reinforced lipase-containing protocells (+Fmoc, P@CV, dark green dots) under high ionic strength. Leakage of calcein into the surrounding solution (+Fmoc, Environment, light green squares) is negligible over the initial 30 min period.

As coacervate droplets prepared by electrostatic complexation are often dissociated under high ionic strength, we designed two on-board enzyme-driven mechanisms based on guest- or host-mediated matrix reinforcement to structurally stabilize the proteinosome-in-coacervate vesicles and thereby extend their functionality under adverse conditions. To implement guest-mediated reinforcement of the coacervate matrix, we first demonstrated that GOx-containing proteinosomes alone could be used as semi-permeable microscale reactors for initiating the reversible assembly of a network of peptide nanofilaments (Figure S13). The addition of aqueous solutions of glucose and the derivatized dipeptide Fmoc-Ala-Ala-OH (Ala, alanine; Fmoc-AA-OH) to proteinosomes at pH 9 resulted in acidification of the proteinosome interior and assembly of a hydrogel network due to protonation of the peptide C-terminal carboxylic acid and onset of supramolecular stacking via hydrophobic and π–π interactions.^57^ LSCM images indicated that the peptide nanofilaments nucleated within the proteinosomes at approximately pH 7 and that further growth gave rise to the hydrogelation of the proteinosomes and their external environment as the pH decreased to around 6 (Figure S13). Given these observations, we prepared GOx/gelatin/proteinosome-in-coacervate droplets at pH 9 and added glucose and Fmoc-AA-OH to simultaneously initiate membranization and peptide assembly, respectively (Figure 2a and Figure S14). Sequestration of Fmoc-AA-OH into the proteinosome-in-coacervate constructs at pH 9 had minimal influence on the morphology of the hybrid droplets (Figure 2b). In contrast, reconfiguration into the corresponding hybrid coacervate vesicles at pH values below 6 was affected by incipient self-assembly of the peptide nanofilaments. Specifically, osmotically induced compression of the alginate/CSF coacervate phase was inhibited such that the membrane domain remained wide enough to house multiple small water vacuoles as well as a large, centralized lumen containing expelled proteinosomes (Figure 2c and Figure S15). Incorporation of the nanofilaments in the coacervate matrix increased the mechanical robustness of the coacervate vesicles (Figure S16) and considerably decreased the fluidity of the constituent coacervate phase as determined by fluorescence recovery after photo-bleaching (FRAP) experiments (Figure 2d and Figure S17). Increasing the pH to 9 regenerated the coacervate droplets, indicating that reinforcement of the coacervate matrix by in situ dipeptide self-assembly was reversible (Figure S18).

Alternatively, we implemented a host-mediated process of matrix reinforcement in the proteinosome-in-coacervate vesicles by replacing alginate with a synthesized tyramine-derivatized alginate (alginate-Tyr; Figure S19) to generate silk-based coacervates capable of post-assembly covalent cross-linking (Figure 2e). Initial experiments indicated that the activation of GOx-containing proteinosomes in the presence of glucose and horseradish peroxidase (HRP) produced extensive hydrogelation in aqueous solutions of alginate-Tyr (Figure S20) and confirmed that alginate-Tyr/CSF positively charged coacervate vesicles and near-neutral coacervate droplets could be readily assembled (Figure S21). Consequently, alginate-Tyr/CSF coacervate vesicles were prepared at pH 4 and subsequently transformed at pH 9 into coacervate droplets with captured urease/GOx/gelatin-containing proteinosomes and sequestered HRP. Addition of glucose and production of gluconic acid and hydrogen peroxide over 12 h resulted in membranization and HRP-mediated oxidative cross-linking of alginate-Tyr, respectively, to produce multi-chambered vesicles comprising a structurally reinforced coacervate matrix infiltrated with multiple proteinosome-containing vacuoles (Figure 2f and Figure S22). No reconfiguration of the coacervate vesicles into coacervate droplets was observed after the subsequent addition of urea and urease-mediated alkalinization of the suspension (Figure 2g and Figure S23), indicating that cross-linking of the alginate-Tyr chains was irreversible under the conditions employed.

Structural reinforcement of the coacervate matrix by Fmoc-AA-OH nanofilament self-assembly or alginate-Tyr crosslinking stabilized the proteinosome-in-coacervate vesicles when exposed to high-ionic strength solutions. The modified constructs remained unchanged in the presence of sodium chloride, while coacervate vesicles prepared without Fmoc-AA-OH or cross-linked constituents disintegrated within 5 min, releasing intact proteinosomes into the external environment (Figure 2h–j and Figure S24). Guest-mediated reinforcement of the coacervate membrane domain with peptide nanofilaments enabled sequestered lipase to remain active within the coacervate vesicles under high-ionic strength conditions (Figure 2k,l).

Taken together, the results indicate that endogenous GOx activity in the guest proteinosomes or a GOx/HRP cascade between the guest and host domains can be used respectively to implement reversible or irreversible structural reinforcement of the hybrid vesicles. In both mechanisms, reinforcement and reconfiguration of the coacervate droplet matrix proceeds in parallel due to the co-utilization of the enzyme-derived outputs (gluconic acid or gluconic acid/hydrogen peroxide) and concurrent processes of membranization, vacuole expansion, dipeptide nanofilament assembly, and alginate-Tyr cross-linking.

### Catalytic Reactivity and Selective Disintegration in Co-Trapped Binary Proteinosome Populations

**Figure 3:**
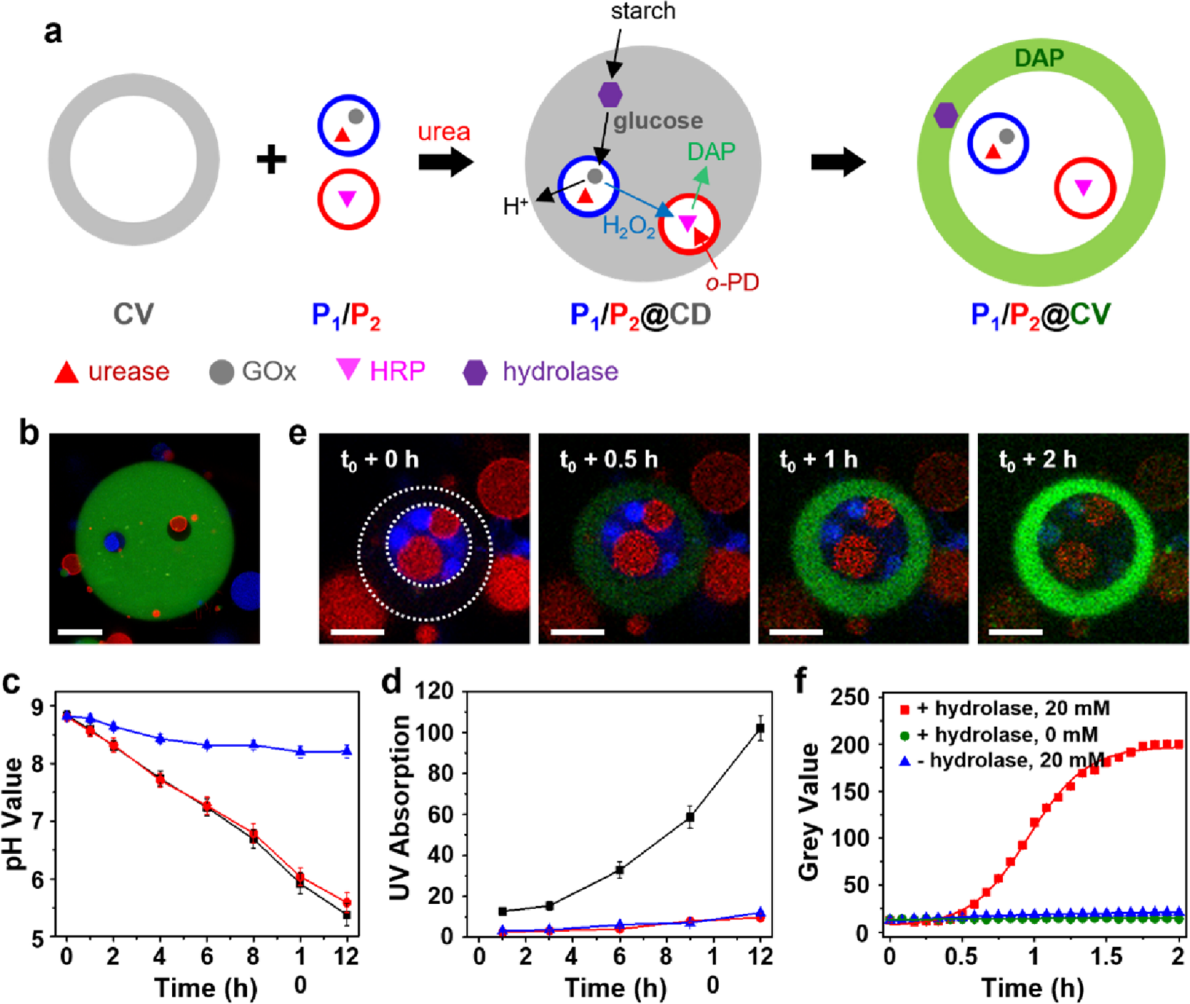
Catalytic reactivity in co-trapped protocell populations. (a) Urease (red triangles)/GOx (gray dots)-containing proteinosomes (P_1_, blue rings) and HRP (pink triangles)-containing proteinosomes (P_2_, red rings) are co-captured within alginate/CSF coacervate vesicles (CV, gray ring) by endogenously controlled enzyme-mediated reconfiguration via a fuel-driven two-step pH-oscillation process involving membrane-less host/guest coacervate droplets (P_1_/P_2_@CD, gray circle) intermediates loaded with starch hydrolases (purple hexagons). Co-production of hydrogen peroxide acts as a diffusive signal to drive *o*PD peroxidation in the captured HRP-containing proteinosomes, leading to a green fluorescence output (2,3-DAP) in the host/guest coacervate vesicles (P_1_/P_2_@CV, green circle). (b) LSCM image of a single P_1_/P_2_@CD showing co-located P_1_ and P_2_ guest protocells. Fluorescent labels: coacervate droplets (FITC-CSF, green); proteinosomes, P_1_ (Dylight 405, blue), P_2_, (RITC, red). Scale bars, 20 μm. (c, d) Time-dependent changes in pH ((c), membranization) and absorbance at 495 nm ((d), 2,3-DAP production, arbitrary units). Plots for P_1_/P_2_@CV after the addition of starch and *o*PD (black squares) show decrease in pH and increase in absorbance. Plots for P_1_@CV (red circles) show a similar decrease in pH (c) but a negligible change in absorbance (d). Plots for P_2_@CV (blue triangles) show negligible changes in pH and absorbance. (e) Time-sequence of fluorescence images of a single P_1_/P_2_@CV before (*t*_0_) and after (*t*_0_ = 0.5, 1, and 2 h) the addition of glucose. Production of 2,3-DAP via an inter-proteinosome GOx/HRP cascade reaction gives rise to green fluorescence in the unlabeled coacervate matrix. Blue and red labels as shown in (b). Scale bars, 20 μm. (f) Time-dependent changes in green fluorescence (2,3-DAP, gray value) recorded on individual coacervate vesicles containing captured GOx- and HRP-loaded proteinosomes after the addition of *o*PD and glucose in the presence of starch and starch hydrolases (red triangles), without starch but with starch hydrolases (green circles), or with starch but without starch hydrolases (blue triangles).

Having established that alginate/CSF coacervate vesicles containing single populations of proteinosomes can be assembled via a sequential mechanism of spontaneous capture and enzyme-mediated membranization, we sought to exploit the methodology for sequestration and chemical processing of co-trapped binary protocell populations (Figure 3a). First, we demonstrated that urease/GOx- and HRP-loaded proteinosomes could be co-captured within starch hydrolase-containing coacervate droplets in the presence of urea (Figure 3b). Fuel-driven membranization was then initiated in the presence of starch and *o*-phenylenediamine (oPD, HRP substrate) such that the reconfiguration mechanism (acidification) was coupled to the catalytic production of 2,3-diaminophenazine (2,3-DAP) (Figure 3c,d and Figures S25 and S26). Increasing starch concentrations increased both the rates of GOx-mediated pH decrease and GOx/HRP-dependent product production (Figure S27), indicating that diffusive transfer of hydrogen peroxide occurred between the co-trapped proteinosomes. Corresponding LSCM images of individual coacervate vesicles confirmed the spatially confined catalytic reactivity of the co-located proteinosomes and the dependence on an inter-protocell hydrogen peroxide signaling pathway (Figure 3e,f).

**Figure 4:**
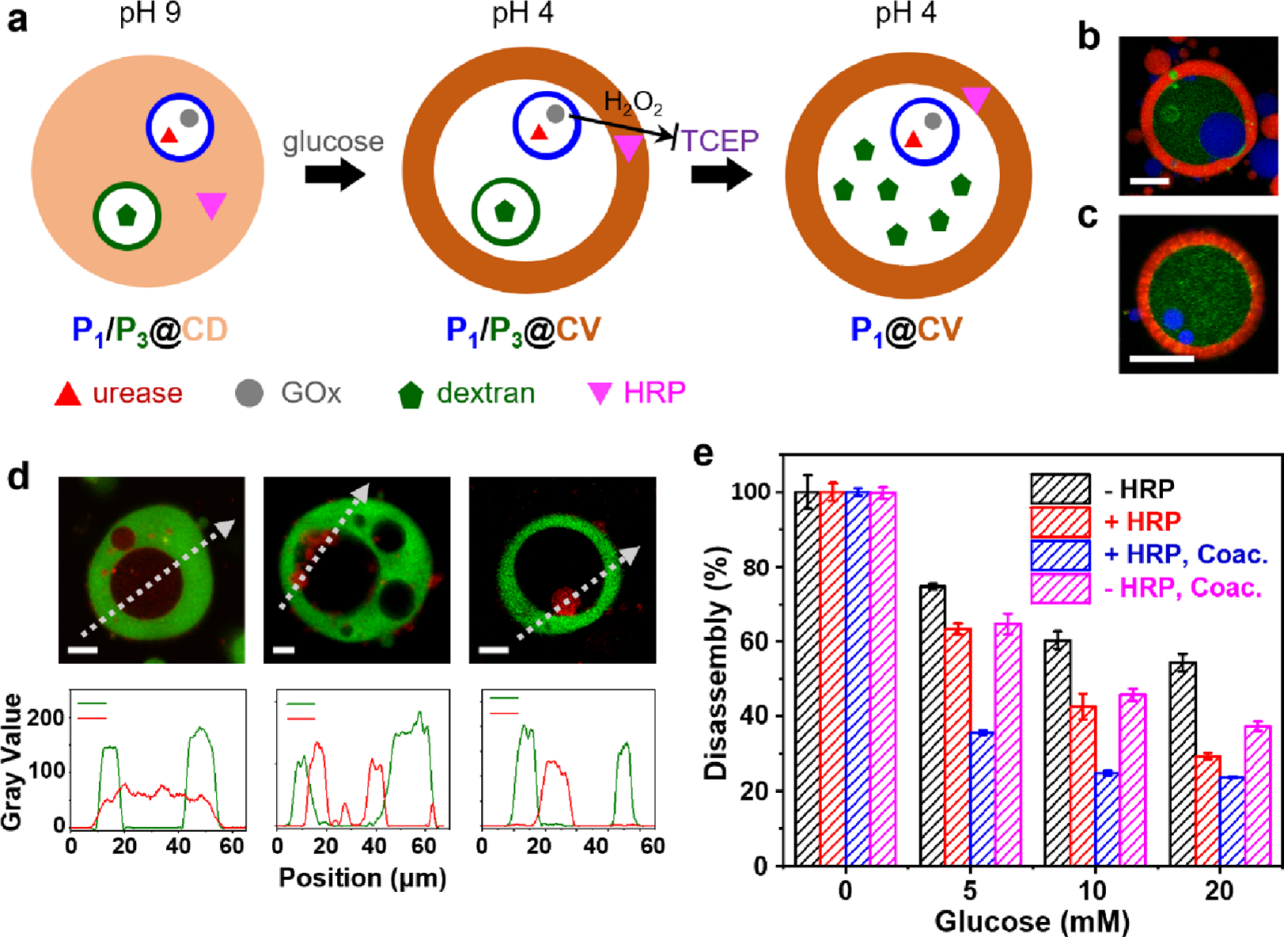
Selective disintegration in co-trapped protocell populations. (a) Urease (red triangles)/GOx (gray dots)-containing proteinosomes with PEG-NHS cross-links (P_1_, blue rings) and dextran-(green pentagons)-containing proteinosomes with PEG-NHS-disulfide cross-links (P_3_, green rings) are co-captured within alginate/CSF coacervate droplets (P_1_/P_3_@CD, light orange circle) at pH 9 and reconfigured at pH 4 into P_1_/P_3_@CV, brown rings). Addition of TCEP results in the selective disassembly of P_3_, formation of P_1_@CV, and release of dextran into the lumen. Simultaneous addition of TCEP and glucose offsets disassembly of P_3_ by on-site production of hydrogen peroxide (thin black arrow); the defense mechanism is further enhanced by sequestration of HRP (pink triangles) in the coacervate scaffold. (b, c) LSCM images showing P_1_/P_3_@CV with co-captured P_1_ (blue, Dylight 405) and P_3_ (green, FITC-dextran) after the addition of TCEP showing partial (b, small green ring) and complete (c) disassembly of P_3_ and concomitant release of FITC-dextran into the lumen containing intact P_1_. The coacervate phase is labeled with RITC-CSF (red fluorescence). Scale bars, 20 μm. (d) LSCM images (top row) and corresponding line profiles (bottom row) of multi/single-chambered coacervate vesicles containing FITC-dextran (green) and captured disulfide-cross-linked proteinosomes containing RITC-gelatin (red) and GOx recorded 8 h after the addition of TCEP (10 mM) in the absence of glucose (left) or simultaneous addition of TCEP and glucose (10 mM (middle), 20 mM (right)). Without glucose, no proteinosomes are observed and the gelatin payload is observed throughout the host/guest protocell. Intact TCEP-sensitive proteinosomes are observed under GOx activity. Scale bars are 20 μm. Dashed white lines indicate the positions of the recorded line profiles (bottom row). (e) Bar chart showing percentage of captured GOx-containing proteinosomes undergoing complete disassembly in the presence of TCEP (5 mM) and various concentrations of glucose. Proteinosomes were captured in the presence (15 IU/mL, red) or absence (black) of HRP-containing alginate/CSF coacervate droplets (blue) or suspended in bulk solution (purple). Data recorded after 6 h.

In a second approach, we used intrinsic differences in membrane stability to chemically disintegrate a selected subset of protocells within a co-captured binary population of proteinosomes (Figure 4a). To achieve this, we prepared two types of negatively charged proteinosomes consisting of cross-linked protein–polymer nanoconjugate membranes that were chemically robust (PEG-NHS cross-linker) or susceptible (PEG-NHS-disulfide cross-linker) to scission in the presence of the reducing agent tris(2-carboxyethyl)phosphine hydrochloride (TCEP).^37^ The former proteinosomes were prepared as above and contained gelatin along with co-encapsulated urease and GOx, while the latter contained gelatin and a macromolecular payload (FITC-labeled dextran, *M*_w_ = ca. 2000 kDa) and were enzymatically inactive. A 1:1 mixture of the two different protocells was prepared, added to a suspension of alginate/CSF coacervate vesicles, and then co-captured using an bi-enzyme-mediated two-step pH-oscillation processing pathway. Selective disassembly of the PEG-NHS-disulfide-cross-linked proteinosomes occurred within the lumen over ca. 2 h by the addition of TCEP, resulting in the release of FITC-dextran into the water-filled vacuole (Figure 4b,c). No disassembly of the coacervate matrix was observed, indicating that the reaction of TCEP with the disulfide cross-links of silk fibroin was negligible under the conditions employed.

Given the above observations, we implemented an endogenous proteinosome-based self-defense mechanism to mitigate TCEP-mediated selective membrane disassembly. To achieve this, we added glucose simultaneously with TCEP to deactivate the reducing agent by reaction with hydrogen peroxide arising from proteinosomes containing GOx. Consequently, an increasing number of the disulfide-crosslinked proteinosomes remained intact in the lumen of the host/guest coacervate vesicles as the glucose concentration was progressively increased (Figure 4d and Figure S28). While the addition of TCEP at concentrations of 10 mM in the absence of glucose led to the disassembly of all the disulfide-cross-linked proteinosomes, the simultaneous addition of glucose and TCEP decreased the percentage of the disassembled proteinosomes over 6 h to approximately 40% at a glucose concentration of 20 mM (Figure 4e). The protocell self-defense mechanism was further enhanced by the incorporation of HRP in the host coacervate phase, which served as a catalyst for TCEP oxidation in the presence of hydrogen peroxide (Figure 4e and Figure S28). Interestingly, non-captured proteinosomes present in the external environment were disassembled under these conditions, indicating that the mitigation of TCEP-induced disassembly was more effective when the defense mechanism was embedded within the coacervate matrix. This was consistent with control experiments undertaken in the absence of a coacervate phase, which showed higher levels of membrane disassembly at the same glucose and TCEP concentrations (Figure 4e), indicating that self-protection of the proteinosome population was enhanced when the reaction system was confined to a molecularly crowded environment. We attributed this to the local accumulation of hydrogen peroxide and HRP around the trapped proteinosomes compared with free diffusion and dilution of the oxidant arising from proteinosomes present in the external solution.

## Conclusions and Outlook

In this paper, we implement the self-driven capture and organization of single or binary populations of proteinosomes within reconfigurable alginate/silk fibroin coacervate vesicles as a step toward the autonomic integration of diverse protocells in nested cytomimetic systems. In contrast to previous studies that determined the experimental conditions responsible for spontaneous membranization in silk-based coacervate protocells,^54^ herein, we develop a novel strategy for the chemically triggered association of different protocell populations via sequential demembranization and membranization of liquid–liquid phase-separated droplets. Our approach is dependent on the self-membranization of silk-based coacervate droplets and involves reversible membrane dynamics and osmotically induced expansion accompanying guest protocell-mediated enzyme activity. Consequently, interchange of the coacervate vesicle and droplet morphologies by fuel-driven reactions produces discrete co-organized protocell communities capable of self-induced matrix reinforcement, integrated catalytic activity, and selective membrane disintegration.

Although capture efficiencies of 62% are achieved in the presence of a large excess of coacervate vesicles, only 30% of the proteinosome population is sequestered at a proteinosome:coacervate vesicle number ratio of 1:2. Thus, under these conditions, both non-captured and captured proteinosomes contribute to the endogenous GOx-mediated decrease in pH associated with reconfiguration of the coacervate droplets into coacervate vesicles (Figure S29). Moreover, changing the spatial location of the enzymes such that GOx/urease-sequestered coacervate droplets are mixed with non-enzymatic proteinosomes has minimal effect on the capture of the proteinosomes after the addition of urea. However, subsequent reconfiguration after the addition of glucose into single-chambered coacervate vesicles is less efficient, producing multi-compartmentalized vesicles in which the proteinosomes remain attached to the coacervate phase (Figure S30), suggesting that the locus of H^+^ production influences the reconfiguration pathway.

It should be possible to develop our approach to the self-driven capture of living cells in coacervate vesicles for the development of nested cellular bionic systems with high biocompatibility. For example, fuel-driven activation of coacervate membranization can be accomplished by capturing live yeast cells within alginate/CSF coacervate droplets containing starch hydrolases and adding starch to the external environment to generate glucose for cellular metabolism and growth (Figure S31). Consequently, the resulting acidification associated with CO_2_ production triggers reconfiguration of the coacervate droplets into stabilized coacervate vesicles, which expels the captured yeast cells into the aqueous-filled lumen where densely packed colonies of viable cells develop over 36 h.

In general, our semi-autonomous mechanism for constructing cell-like nested communities is inspired by the notion of endosymbiosis^58^ and offers a step to self-driven artificial multicellularity in life-like materials. The development of reconfigurable cytomimetic materials with structural, functional, and organizational complexity could provide future opportunities for bottom-up synthetic biology, soft microbotics, and programmable storage–release technology.

## Supporting Information Available

Experimental materials and methods; microscopy images; SEM images; FACS characterization of protocell populations; zeta potential of coacervate microdroplets/vesicles; FTIR spectra of polymers; UV/vis absorption spectra (PDF)
