# Supplementary material for: Autonomic Integration in Nested Protocell Communities
Source: J Am Chem Soc. 2023 Jun 27;145(27):14727–36. doi: 10.1021/jacs.3c02816 (PMC10347549; doi:10.1021/jacs.3c02816)
Supplement: Supplementary file 1 — ja3c02816_si_001.pdf [file ja3c02816_si_001.pdf]

Autonomic integration in nested protocell communities

Zhuping Yin[1], Ning Gao[1,2], Can Xu[1], Mei Li[1,3]* and Stephen Mann[1,2,3,4]*

[1] *Centre for Protolife Research, School of Chemistry, University of Bristol, Bristol. BS8 1TS, UK.*
[2] *Max Planck-Bristol Centre for Minimal Biology, School of Chemistry, University of Bristol, Bristol BS8 1TS, UK.*
[3] *School of Materials Science and Engineering, Shanghai Jiao Tong University, Shanghai 200240, P. R. China.*
[4] *Zhangjiang Institute for Advanced Study (ZIAS), Shanghai Jiao Tong University, 429 Zhangheng Road, Shanghai 201203, P. R. China.*

Supplementary Information

1. Supplementary Materials and Methods (Page 1-Page 9)

2. Supplementary Figures (Page 10-Page 29)

1. Supplementary Materials and Methods

Materials

The following chemicals were used as received: alginic acid sodium salt (Sigma-Aldrich, Mw: 140-160 kDa), gelatin from porcine skin (Type A, Sigma-Aldrich), bovine serum albumin (BSA, Sigma-Aldrich, heat shock fraction, > 98%), starch (soluble, Sigma-Aldrich), peptone (Scientific Laboratory Supplies), yeast extract (VWR), dextran (Mw: *ca*. 250 kDa, Sigma-Aldrich), FITC-dextran (Mw: *ca*. 250 kDa, Sigma-Aldrich), glucose oxidase from *Aspergillus niger* (GOx, Type X-S, lyophilized powder, 100,000-250,000 units/g solid, Sigma-Aldrich), urease from *Canavalia ensiformis* (*ca.* 8 U/mg, Sigma-Aldrich), peroxidase from horseradish (*ca.* 150 U/mg, Sigma-Aldrich), α-amylase from porcine pancreas (Type I-A, ≥1000 units/mg protein, Sigma-Aldrich), amyloglucosidase from *Aspergillus niger* (*ca*. 120 U/mg, Sigma-Aldrich), fluorescein isothiocyanate isomer I (FITC, ≥ 90%, Sigma-Aldrich), rhodamine B isothiocyanate (RITC, Sigma-Aldrich), DyLight 405 NHS Ester (Thermo Fisher Scientific), SYTO 9 (Fisher Scientific Ltd), propidium iodide (PI, Thermo Fisher Scientific), lithium bromide (anhydrous, Sigma-Aldrich), 1,6-hexanediamine (98%, Sigma-Aldrich), 1-ethyl-3-(3-dimethylaminopropyl)carbodiimide (EDC, Thermo Fisher Scientific), D-(+)-glucose (≥ 99.5%, Sigma-Aldrich), urea (BioReagent, Sigma-Aldrich), O,O′-bis[2-(N-succinimidyl-succinylamino)ethyl]polyethylene glycol (PEG-NHS, Mw: 2000, Sigma-Aldrich), 4,7,10,13,16,19,22,25,32,35,38,41,44,47,50,53-hexadecaoxa-28,29-dithiahexapentacontanedioic acid di-N-succinimidyl ester (PEG-NHS-DS, Sigma-Aldrich), tris(2-carboxyethyl)phosphine hydrochloride (TCEP, VWR International), *o*-phenylenediamine (oPD, ≥ 98.0%, Sigma-Aldrich), L-alanine, N-[(9H-fluoren-9-ylmethoxy)carbonyl]-L-alanyl-(2S)-2-[[(2S)-2-(9H-fluoren-9-ylmethoxy carbonylamino)propanoyl]amino]propanoic acid (Fmoc-Ala-Ala-OH; Insight Biotechnology).

Preparation of regenerated silk fibroin

B. mori cocoons were boiled in 0.05% Na_2CO_3 solution at 98-100 ^oC for 30 min and rinsed with deionized water (DI water) to discard surface-attached sericin. The procedure was then repeated for two more times. Degummed silk fibers were then transferred into an oven and dried (60 ^oC, 6 h). Regenerated mulberry silk fibroin (SF) solution was prepared by dissolving the degummed/dried silk fibers in aqueous lithium bromide (LiBr, 9.3 M, 60 ^oC, 1 h) under stirring and the solutions then cooled to room temperature[1].

Synthesis and characterization of cationized silk fibroin

Cationized silk fibroin (CSF) was prepared by following a previously reported procedure.[2] Briefly, 1,6-hexanediamine (3000 mg) was dissolved in 3 ml DI water and the pH adjusted to 6.5 using NaOH/HCl. The 1,6-hexanediamine solution was then added to a cooled SF solution (150 mg/mL, LiBr/H_2O, 6 mL) under stirring, followed by addition of EDC (200 mg) to start the amination reaction. The reaction pH was maintained at 6.5 by repeated additions of NaOH/HCl once every hour for 6 h. A final aliquot of EDC (200 mg) was then added in the last pH manipulation and the mixture then left under stirring at room temperature overnight. The produced CSF was then purified by dialysis against DI water for 3-4 days using a cellulose dialysis tube (Sigma, MWCO: 12-14 kDa) and then filtered using a MF-Millipore membrane (Sigma, 0.45 µm). The concentration of the purified CSF solution was determined by weighing the solution (1 ml) before and after lyophilization. Fluorescence labelling was implemented by addition of FITC, RITC or Dylight 405 (75 µL, 2 mg/mL, DMSO) to the dissolved SF solution (LiBr/H_2O, 6 ml) under stirring at room temperature over 5 h prior to the amination reaction. Zeta potential

measurements were used to characterize the charge of the silk-based polymers (CSF and SF, 1 mg/ml) using a zeta potentiometer analyser (Malvern Instruments, UK).

The primary amino content in different silk-based polymers was determined using TNBSA (2,4,6-trinitrobenzene sulfonic acid), which reacts with primary amino group (-NH_2) to generate a coloured product.[2] Typically, CSF and SF were separately diluted to 0.2 mg/ml in Na_2CO_3/$NaHCO_3$ buffer (0.1 M, pH 8.5), followed by additions of 0.25 ml TNBSA (0.05%, w/v, buffer 8.5) to each group (0.5 ml) and incubation at 37 ^oC for 2 h. 0.25 ml HCl (0.5 M) and 0.5 ml SDS were then added to each sample to stop and stabilize the reaction before analysis by UV/VIS spectroscopy (250-700 nm, Lambda 35, PerkinElmer, USA). Using the above protocol, BSA (0.05, 0.1, 0.15, 0.2 mg/ml) was utilized as a standard reagent to produce a calibration curve (absorption at 335 nm vs -NH_2 content) to determine the primary amino content in the CSF or SF polymers.

Preparation and characterization of alginate/CSF coacervates

Silk-based coacervates were produced via an electrostatically mediated associative liquid-liquid phase separation in mixtures of positively charged CSF and negatively charged alginate (Mw:140-160 kDa). The mixtures were prepared at room temperature at different charge ratios. The alginate [COOH] : CSF [NH_2] charge ratio was estimated as the ratio of alginate monomer concentration (x1 COOH per monomer) to the primary amino concentration of CSF determined by TNBSA analysis. The charge ratio was controlled by compositional changes of [COOH] (alginate) and [NH_2] (CSF) or via pH manipulation through additions of NaOH or HCl.

Typically, positively charged coacervate vesicles (CV), positively charged multi-compartmentalized coacervate droplets (MCV) and neutral coacervate droplets (CD), were prepared by increasing the volume of a stock alginate solution ([COOH] 100 mM, 0.5-20 µl) added to a stock CSF solution ([NH_2] 8.98 mM, 11-67 µl) under stirring, followed by additions of different amounts of H_2O to make up the total volume to 100 µl for each sample. The final [COOH] (alginate) concentrations were 0.25-20 mM and the [COOH] (alginate) : [NH_2] (CSF) ratios were between 0.05-3. Alternatively, different microstructures were produced by increasing the volume of added alginate ([COOH] 100 mM, 2-8 µl) to stock CSF solutions ([NH_2] 8.98 mM, 22-88 µl) followed by additions of different amounts of H_2O to make up the total volume to 100 µl for each sample to give final [COOH] (alginate) and [NH_2] (CSF) concentrations of 2/2, 4/4, 6/6 or 8/8 mM. Stoichiometric aliquots of NaOH or HCl were then added to each group to control the pH from pH 3 to 9. The silk-based coacervate microstructures were imaged using a Leica DMI3000 B fluorescence microscope (Leica, Germany) and/or a SP5-II confocal laser scanning microscope (LSCM, Leica, Germany) and analysed using Image J software. Zeta potential distributions were used to characterize the surface charge of the different alginate/CSF microstructures (Malvern Instruments, UK).

Preparation of proteinosomes

Synthesis of PNIPAAm polymer, preparation of BSA/PNIPAAm nanoconjugates and assembly of proteinosomes were achieved by following a reported procedure[2]. Typically, an aqueous mixture (40 µl) comprising BSA/PNIPAAm nanoconjugates (30 mg/ml, 15 µl), Na_2CO_3/$NaHCO_3$ (100/100 mM, pH 8.5, 15 µl) and dissolved PEG-NHS (cross-linker, 2.5 mg, 10 µl) were mixed with 650 µl 2-ethyl-1-hexanol (oil phase) under mechanical disturbance to produce a dispersed water-in-oil Pickering emulsion droplets. The emulsion was left at 4 ^oC for 2 days to stabilize and precipitate the emulsion droplets. The precipitated emulsion droplets were then transferred into 1 ml of 70 % ethanol after removal of the oil supernatant, followed by centrifugation at 10000 rpm for 5 min to precipitate the

proteinosomes and removed the supernatant. Redispersion and centrifugation of the proteinosomes were then repeated twice using 1 ml 40 % ethanol and then DI water. The proteinosome aggregate was then dispersed into 300 µl DI water and stored at 4 ^oC. If required, proteinosomes with smaller size could be produced by sonicating the emulsion droplets in an ultrasonic bath at 300 W for 1 min.

Proteinosomes with encapsulated biomolecules (gelatin, GOx, urease) were prepared by dissolving the guest components in aqueous solution at concentrations of 10, 4 and 4 mg/ml, respectively, prior to emulsification. Briefly, an aqueous solution (40 µl) comprising BSA/PNIPAAm nanoconjugates (30 mg/ml, 15 µl), RITC-gelatin (60 mg/ml, $Na_2CO_3/NaHCO_3$ 100/100 mM, pH 8.5, 7 µl), GOx (40 mg/ml, $Na_2CO_3/NaHCO_3$ 100/100 mM, pH 8.5, 4 µl), urease (40 mg/ml, $Na_2CO_3/NaHCO_3$ 100/100 mM, pH 8.5, 4 µl) and dissolved PEG-NHS (cross-linker, 2.5 mg, 10 µl), were mixed with 650 µl 2-ethyl-1-hexanol (oil phase) under sonication (Ultrasonic Bath 300 W) for 1-2 min to produce discrete water-in-oil Pickering emulsion droplets. The emulsion system was processed as above to produce water-in-water proteinosomes, which were the characterized using a Leica DMI3000 B fluorescence microscope (Leica, Germany) and/or a SP5-II confocal laser scanning microscope (LSCM, Leica, Germany) and analyzed with Image J software.

Proteinosomes with membranes susceptible to disassembly when in the presence of reducing reagents (TCEP, DTT, L-Cysteine, etc.) were prepared by following the above-mentioned procedure but instead of PEG-NHS, a disulfide-containing cross-linker susceptible to reductive cleavage (PEG-NHS-DS) was used. Typically, an aqueous solution (40 µl) consisting of BSA/PNIPAAm nanoconjugate (30 mg/ml, 15 µl), RITC-gelatin (60 mg/ml, $Na_2CO_3/NaHCO_3$ 100/100 mM, pH 8.5, 4 µl), $Na_2CO_3/NaHCO_3$ buffer (100/100 mM, pH 8.5, 11 µl) and PEG-NHS-DS (0.27 mg, 10 µl) were dispersed into 650 µl 2-ethyl-1-hexanol (oil phase) under sonication (ultrasonic bath, 300 W) for 1-2 min. The emulsion was then left at 4 ^oC for 2 days. The produced proteinosomes were then purified and transferred into aqueous phase by following the above methods.

Self-driven construction of proteinosome-in-coacervate vesicles

Alginate/cationized silk fibroin coacervate vesicles with captured proteinosomes were constructed using a two-step procedure involving (1) the uptake of urease/GOx/gelatin-containing proteinosomes into membrane-less coacervate droplets by endogenous urease-mediated reconfiguration of positively charged coacervate vesicles, and (2) reconfiguration of the proteinosome-in-coacervate droplets into proteinosome-in-coacervate vesicles by endogenous GOx activity. In step 1, a population of proteinosomes containing urease and GOx (4 x 10^7/ml, 50 µl) was mixed with positively charged coacervate vesicles ([NH_2] 8 mM, [COOH] 8 mM, 100 µl, pH = 6) at an approximate proteinosome : vesicle number ratio of 1 : 2. Urea was then added (500 mM, 8-24 µl), followed by addition of different volumes of DI water to make up the total volume to 200 µl. The mixture was then incubated at room temperature for 6-8 hours to produce coacervate droplets containing capture proteinosomes. In step 2, glucose (500 mM, 8-24 µl) was added to the suspension of proteinosomes-in-coacervate droplets ([NH_2] 4 mM, [COOH] 4 mM, pH ~ 9.0) and the suspension left at room temperature for 6-8 h.

The number densities present in single populations of the coacervate vesicles and proteinosomes were determined using LSCM and Image J software. The number of protocells in a unit volume were counted. Typically, when mixed, the proteinosome and coacervate vesicle final number densities were approximately 1 x 10^7/ml and 2 x 10^7/ml, respectively. Thus, experiments involving the capture of proteinosomes in positively charged coacervate vesicles were undertaken at an approximate proteinosome : vesicle number ratio of 1 : 2.

Changes in pH were monitored using a pH meter (METTLER TOLEDO, Switzerland) controlled by Lab X software. LSCM was used to record the formation and reconfiguration of the hybrid protocells protocell at different pH values. FACS analyses were undertaken on single populations of positively charged coacervate vesicles ([NH_2] 4 mM, [COOH] 4 mM, pH *ca.* 6.0), membrane-less coacervate droplets ([NH_2] 4 mM, [COOH] 4 mM, pH *ca.* 9.0) or enzyme-containing proteinosomes. FACS analyses were also undertaken on binary populations of positively charged vesicles and proteinosomes before and after the stepwise addition of urea (30 mM) and glucose (60 mM). All investigations used a FACS Canto II flow cytometer operating at a low pressure with a 100 µm sorting nozzle. 2D pseudo-color plots of the FSC-A and SSC-A light were determined for a total of 20,000 particles in the single or binary populations. Data analysis was performed on FlowJo 7.6 software.

Fuel-driven reconfiguration in guest/host protocells.

GOx/gelatin-loaded proteinosomes (GOx, 4 mg/ml, 1 x 10^7/ml) were captured in alginate/CSF coacervate droplets ([NH_2] 8 mM, [COOH] 8 mM, RITC-CSF, 100 µl) containing a sequestered mixture of amylase and amyloglucosidase (40/40 IU/ml). Soluble starch was then added (500 mM, 0-24 µl). Changes in pH were recorded at different time points (0-12 h) and corresponding LSCM images of the reconfiguration process recorded and analyzed using Image J software.

Glucose production by enzymatic hydrolysis of soluble starch

An integrated enzyme system comprising α-amylase and amyloglucosidase was used to efficiently hydrolyze soluble potato starch to glucose. Amylase was used to hydrolyse starch polymers to maltose, maltotriose, dextrin and minimal amounts of glucose, while amyloglucosidase was included to hydrolyse the maltose/maltotriose/dextrin products to glucose.

Test experiments to assess the efficiency of starch hydrolysis were undertaken as follows. A concentrated starch solution (500 mM, monomer concentration) was prepared by dissolving potato starch in DI water under stirring and leaving the solution at 80 ^{o}C for 2 h. The starch solution was then diluted to 0-90 mM by adding aliquots of the solution (500 mM, 0-18 µl) to different volumes of DI water to make up the total volume to 90 µl. An amylase/amyloglucosidase solution (10 µl, 200/200 IU/ml) was then added to each sample and the mixtures incubated at 25 ^{o}C for 0-24 h to enable starch hydrolysis and glucose production. The reaction was stopped at various times by denaturing the integrated enzymes with 50 µl NaOH (1 M), followed by sonication for 10 min and then addition of 50 µl HCl (1 M). The supernatants were collected by centrifugation at 10,000 rpm for 5 min and the precipitates discarded. The supernatants were then diluted 10-times with DI water and if required stored at room temperature. A mixture comprising 100 µl oPD (100 mM), 100 µl GOx (10,000 IU/ml), 100 µl HRP (200 IU/ml) and 400 µl PBS buffer (pH 6.0) was added to the supernatants (100 µl) and the reaction mixtures incubated at 37 ^{o}C for 30 min. The production of 2,3-DAP from the GOx/HRP enzyme cascade was stopped at different times by addition of 200 µl HCl solution (3 M), followed by 5-times dilution. Glucose concentrations were quantitively measured by UV/VIS absorption spectroscopy using the 2,3-DAP absorbance at 490 nm. Standard plots obtained from known glucose concentrations (0-0.2 mM) were used to calibrate the GOx/HRP assay.

Determination of partitioning for starch and starch hydrolases

Soluble starch (500 mM, 1 µl), amylase (10 mg/ml, 1 µl) or amyloglucosidase (10 mg/ml, 1 µl), were separately mixed with alginate/CSF coacervate droplets (200 µl, [NH_2] 4 mM, [COOH] 4 mM, pH 4). Partition constants were obtained by calculating the ratio of red fluorescence intensity (grey value)

inside and outside individual coacervate droplets imaged by LSCM images and analysed using Image J software. Transformation of the loaded hybrid droplets to positively charged coacervate vesicles was undertaken by addition of aqueous NaOH (0.05 M) to increase the pH to *ca.* 9.0. The distribution of starch and the starch hydrolases in the coacervate vesicles was then characterized by LSCM.

Guest-mediated peptide assembly in proteinosome-in-coacervate vesicles

Fmoc-Ala-Ala-OH was used as a dipeptide capable of self-assembly into a nanofilamentous hydrogel network by hydrophobic interactions and π-π stacking in acid conditions[3]. In test experiments, GOx/gelatin-containing proteinosomes (Dylight 405-labelled, GOx 4 mg/ml) were prepared as described above and dispersed into aqueous Na_2CO_3/$NaHCO_3$ buffer (10/10 mM, pH 8.5, 100 µl) at a number density of 2 x 10^7/ml. The proteinosome suspension was then mixed with aqueous Fmoc-AA-OH solution (20 µl, 10 mg/ml, Na_2CO_3/$NaHCO_3$ buffer, 100/100 mM, pH 8.5) containing dispersed Nile red (0.001 mg/ml, 60 µl Na_2CO_3/$NaHCO_3$ buffer (10/10 mM, pH 8.5)) and peptide self-assembly initiated by addition of glucose solution (20 µl, 400 mM). Changes in pH with time were detected using a pH meter (METTLER TOLEDO, Switzerland) at 4, 12 and 24 h and fluorescence images recorded by LSCM.

Proteinosome-in-coacervate droplets (100 µl, RITC-CSF, [NH_2] 8 mM, [COOH] 8 mM, pH 9.0, proteinosome number density, 2 x 10^7/ml) were prepared in the presence (1.5 mg/ml) or absence (0 mg/ml) of Fmoc-AA-OH. Glucose was then added (0-24 µl, 500 mM) to the suspension, followed by different amounts of DI water to make the total volume up to 200 µl in each group of experiments. Samples were then incubated at room temperature for up to 12 hours and the pH monitored at different time points (0-12 h) and images recorded by LCSM and SEM (Jeol IT300 SEM, Japan). SEM samples were prepared by mounting a drop (10 µl) of the suspensions (10 times diluted) onto the surface of PEG functionalized cover slips and then drying in a N_2 flow for 15 min, followed by a coating with silver prior to SEM imaging.

Changes in the fluidity of the membrane domain in proteinosome-in-coacervate vesicles containing Fmoc-AA-OH filaments were determined by FRAP experiments. Briefly, the dynamic fluorescence recovery of the coacervate matrix was measured using time-sequence LSCM images (over 25 min) of the samples after photo-bleaching localized regions of the host coacervate phase. Corresponding LSCM images were analyzed using Image J software. Similar experiments were done using the unmodified coacervate vesicles containing proteinosomes as well as proteinosome-in-coacervate droplets with or without sequestered Fmoc-AA-OH.

The structural stability of the proteinosome-in-coacervate vesicles after infiltration with Fmoc-AA-OH filaments was investigated by exposing the hybrid protocells to a NaCl diffusion gradient (4 M). The NaCl gradient was generated by adding aqueous NaCl (10 µl, 4 M) to one end of a circular glass channel containing a suspension of the proteinosomes-in-coacervate vesicles (50 µl). Time-dependent LSCM images were recorded and Image J software used to evaluate the time-dependent changes in fluorescence intensities (grey value) of the host coacervate phase and surrounding water-filled environment. Similar experiments were done using the unmodified coacervate vesicles containing proteinosomes as well as proteinosome-in-coacervate droplets with or without sequestered Fmoc-AA-OH.

Retention of enzyme activity in the Fmoc-AA-OH modified proteinosome-in-coacervate vesicles under high ionic strength was investigated by monitoring the lipase-mediated hydrolysis of non-fluorescent calcein-AM to produce a calcein green fluorescence output. Fmoc-AA-OH-containing proteinosome-in coacervate vesicle suspensions (200 µl, [NH_2] 4 mM, [COOH] 4 mM) were prepared

as above but with the inclusion of an acid-resistant lipase (0 or 1 µl, > 100 LCLU/g). The samples were placed in a circular glass channel and a drop (30 µl) of an aqueous solution containing calcein-AM (*ca.* 5.0 x 10^{-2} mM) and NaCl (2.3 M) added at one end to generate a chemical diffusion gradient. Time-dependent images of the hybrid protocells were recorded over 0-8 h by LSCM.

Host-mediated matrix reinforcement in proteinosome-in-coacervate vesicles

Tyramine-functionalized alginate (alginate-Tyr) was synthesized by an EDC/NHS-activated amination reaction. Briefly, 200 mg of sodium alginate and 500 mg of Tyramine · HCl were blended and then dissolved in 10 ml MES buffer (0.2 M, pH 4.7). The reaction was then started by addition of 160/120 mg of EDC/NHS. The reaction mixture was left at room temperature and under stirring for 24 hours and the alginate-Tyr product purified by dialysis against DI water for 3-4 days using a cellulose dialysis tube (Sigma, MWCO: 12-14 kDa) and then lyophilized. The product was characterized using FI-TR spectroscopy (4000-550 cm^{-1}, PerkinElmer, USA) and UV/VIS spectroscopy (250-700 nm, Lambda 750, PerkinElmer, USA).

As a test experiment, a suspension of GOx-containing proteinosomes (4 mg/ml, 4 x 10^7/ml, 100 µl) and HRP solution (200 IU/ml, 0 or 15 µl) were added to an alginate-Tyr solution (10 mg/ml, 200 ul), followed by addition of different amounts of DI water to give a final volume of 400 µl. Gelation was then achieved by addition of 12 µl glucose solution (1 M) to the mixture and an incubation at room temperature for 0-6 hours. UV/VIS absorption spectra of the reaction mixture was collected at different time points (0-6 h). Gelation was estimated by inversion of a glass vial containing this reaction mixture.

Host-mediated matrix reinforcement of proteinosome-in-coacervate vesicles was undertaken as follows. Positively charged coacervate vesicles were prepared by mixing aqueous solutions of RITC-CSF ([NH_2] 8 mM) and alginate-Tyr) ([COOH] 10 mM) at pH 4.0 (total volume, 100 µl). Urease/GOx/gelatin-containing proteinosomes (GOx 4 mg/ml, 4 x 10^7/ml, 50 µl) were then added at an approximate proteinosome : vesicle number ratio of 1 : 2, followed by addition of different volumes of RITC-labelled HRP solution (0-20 µl, 200 IU/ml) and DI water to give a final volume of 200 µl and the pH raised to 9.0 to capture the proteinosomes. Glucose was then added (1.5 M, 4 µl) and the samples incubated at room temperature for 12h. GOx-mediated acidification transformed the coacervate droplets into vesicles while GOx-induced production of hydrogen peroxide gave rise to crosslinking of alginate-Tyr chains in the coacervate matrix. LSCM was used to image the samples before and after addition of glucose. Images were analysed by using Image J software.

The structural stability of the proteinosome-in-coacervate vesicles after alginate-Tyr crosslinking was monitored by LSCM using the following procedures; (i) incubation of the samples for 3 h at room temperature at pH 9 (NaOH) or 8.7 (urease/urea), and (ii) placing the samples in a circular glass tube and exposing the protocells to a diffusion gradient of aqueous NaOH (25 mM) or aqueous sodium chloride (3 M) at pH 4 as described above.

Fuel-driven three-enzyme network in proteinosome-in-coacervate vesicles

Alginate/CSF coacervate droplets ([NH_2] 8 mM, [COOH] 8 mM, RITC-CSF, 100 µl) containing a sequestered amylase/amyloglucosidase mixture (40/40 IU/ml) along with captured GOx-containing proteinosomes ([GOx] = 4 mg/ml, number density = 1 x 10^7/ml) and HRP-containing proteinosomes ([HRP], 4 mg/ml, number density = 1 x 10^7/ml) were prepared at pH 9. Soluble starch (500 mM, 0-24 µl) and oPD (300 mM, 20 µl) were then added followed by different amounts of DI water to make up the total volume to 200 µl in each group. The samples were then incubated at room temperature for

12 h and the pH recorded at various time intervals (METTLER TOLEDO, Switzerland). 10 µl of the reaction mixture was taken at different time points (1, 3, 6 h) and then diluted with 890 µl DI water, followed by addition of 100 µl HCl (3 M) to quench oPD oxidation. The stabilized reaction mixtures were then centrifugated at 10,000 rpm for 5 min to remove any precipitates prior to the UV/VIS absorption measurement. The 2,3-DAP output from the starch hydrolase/GOx/HRP enzyme cascade was measured over time by monitoring the intensity of the absorption band spectroscopy at 490 nm. Time-dependent LSCM images and line profiles were recorded on individual guest/host protocells undergoing 2,3-DAP production.

Control experiments involving the addition of soluble starch (40 mM) to coacervate droplets with single or double populations of GOx- and HRP-containing proteinosomes prepared in the absence of amylase/amyloglucosidase were also undertaken. Test experiments were initially undertaken on coacervate-free suspensions of binary proteinosome populations as follows. A mixture of amylase/amyloglucosidase (200/200 IU/ml, 20 µl) and oPD (300 mM, 20 µl) was added to a binary population of GOx- and HRP-containing proteinosomes in aqueous solution (100 µl; number ratio, 1:1; number density, 2 x 10^7/ml). Aliquots of soluble starch were added (500 mM, 0-24 µl) followed by different amounts of DI water to make up the total volume to 200 µl in each group an incubation at room temperature for 6 hours. Control experiments involving the addition of soluble starch (16 µl, 500 mM, total volume, 200 µl) to single or double populations of GOx- and HRP-containing proteinosomes prepared in the absence of amylase/amyloglucosidase were also undertaken.

TCEP-induced proteinosome disassembly

GOx/RITC-gelatin-containing proteinosomes were prepared using PEG-NHS-DS crosslinker as described above. To achieve membrane disassembly, glucose (0 or 20 mM) and HRP (0 or 15 IU/ml) were added to a suspension of the enzyme-containing proteinosome (number density, 1.6 x 10^4/ml, 190 µl), followed 30 min later by addition of TCEP (100 mM, 10 µl, pH 9.0). The samples were incubated at room temperature for 2 hours. Proteinosome disassembly was monitored at different time points (30, 60 and 120 min) by fluorescence microscopy (Leica DMI3000 B fluorescence microscope.

Disassembly of the proteinosomes (Dylight 405-labelled, GOx 4 mg/ml, gelatin 6 mg/ml) arising from the competition between TCEP reduction of the protein-polymer membrane and TCEP inactivation by GOx-derived H_2O_2 production was quantitively measured by detecting the appearance of blue fluorescence (excitation; 400 nm, emission, 410-650 nm; 5 nm slits) in the continuous phase using a FluoroMax-4 Spectrofluorometer (HORIBA Scientific, Japan).

The level of proteinosome disassembly was determined by addition of different amounts of glucose (200 mM, 0-20 µl), HRP (300 IU/ml, 0 or 10 µl) and TCEP (400 mM, 2.5 µl) to proteinosome suspensions (100 µl, number density, 3 x 10^4/ml), followed by addition of different volumes of DI water to make up the total volume to 200 µl, and incubation at room temperature for 6 hours. The incubated mixtures were then diluted with 200 µl NaCl solution (1 M, coacervate disassembly) and centrifuged at 10,000 rpm for 10 min to discard the precipitates prior to the detection of fluorescence emission in the supernatant. Three parallel samples were used in each group for statistical analysis. Intrinsic background fluorescence ($\mathbf{I_b}$) was determined by monitoring the incubated proteinosome population in the absence of TCEP as a control group.

The percentage of proteinosome disassembly was determined by using the followed equation:

$$Proteinosome\ disassembly\ (\%) = \frac{I_i - I_b}{I_0 - I_b} \times 100\%$$

where $\mathbf{I_i}$ and $\mathbf{I_0}$ are the intensity of fluorescence emission (430 nm) of samples in the presence and absence of glucose addition.

TCEP-induced disassembly of guest proteinosomes in hybrid coacervate droplets and vesicles

A suspension of GOx/gelatin/proteinosomes-in-coacervate droplets ([NH_2] 4 mM, [COOH] 4 mM; GOx, 4 mg/ml; Dylight 405 labelled-proteinosomes, number density, 1 x 10^7/ml; pH ~ 9.0) was prepared. HRP (0 or 10 µl, 300 IU/ml) was then sequestered into the coacervate phase, and the samples incubated at room temperature for 15 min. Disassembly of the guest proteinosomes was started by addition of TCEP (2.5 µl, 400 mM, pH ~ 9.0) in the absence or presence of added volumes of glucose (500 mM, 0-8 µl), followed by different additions of DI water to make up the total volume to 200 µl in each group and incubation at room temperature for 6 h. LSCM used to record images of the coacervate droplet to vesicles reconfiguration and disintegration of the guest proteinosomes 6 h after addition of glucose. Guest proteinosome disassembly was also quantitively characterized by measuring the changes in Dylight 405 blue fluorescence emission in the aqueous continuous phase. Typically, the supernatants of the above samples were collected by centrifugation at 10,000 rpm for 5 min after a dilution with 200 µl NaCl solution (1 M) for coacervate disassociation. Supernatants were examined using a FluoroMax-4 Spectrofluorometer (HORIBA Scientific, Japan; excitation 400 nm, emission 410-650 nm) and the percentage of percentage of proteinosome disassembly calculated from equation 1.

References:

(1) Koh, L.-D.; Cheng, Y.; Teng, C.-P.; Khin, Y. W.; Loh, X. J.; Tee, S. Y.; Low, M.; Ye, E.; Yu, H. D.; Zhang, Y. W. Structures, mechanical properties and applications of silk fibroin materials. *Prog. Polym. Sci.* 2015, 46, 86-110.

(2) Huang, X.; Li, M.; Green, D. C.; Williams, D. S.; Patil, A. J.; Mann, S. Interfacial assembly of protein–polymer nano-conjugates into stimulus-responsive biomimetic protocells. *Nat. Commun.* 2013, 4, 1-9.

(3) Rodon Fores, J.; Martinez Mendez, M. L.; Mao, X.; Wagner, D.; Schmutz, M.; Rabineau, M.; Lavalle, P.; Schaaf, P.; Boulmedais, F.; Jierry, L. Localized supramolecular peptide self-assembly directed by enzyme-induced proton gradients. *Angew. Chem.* 2017, 129, 16200-16204.

2. Supplementary Figures

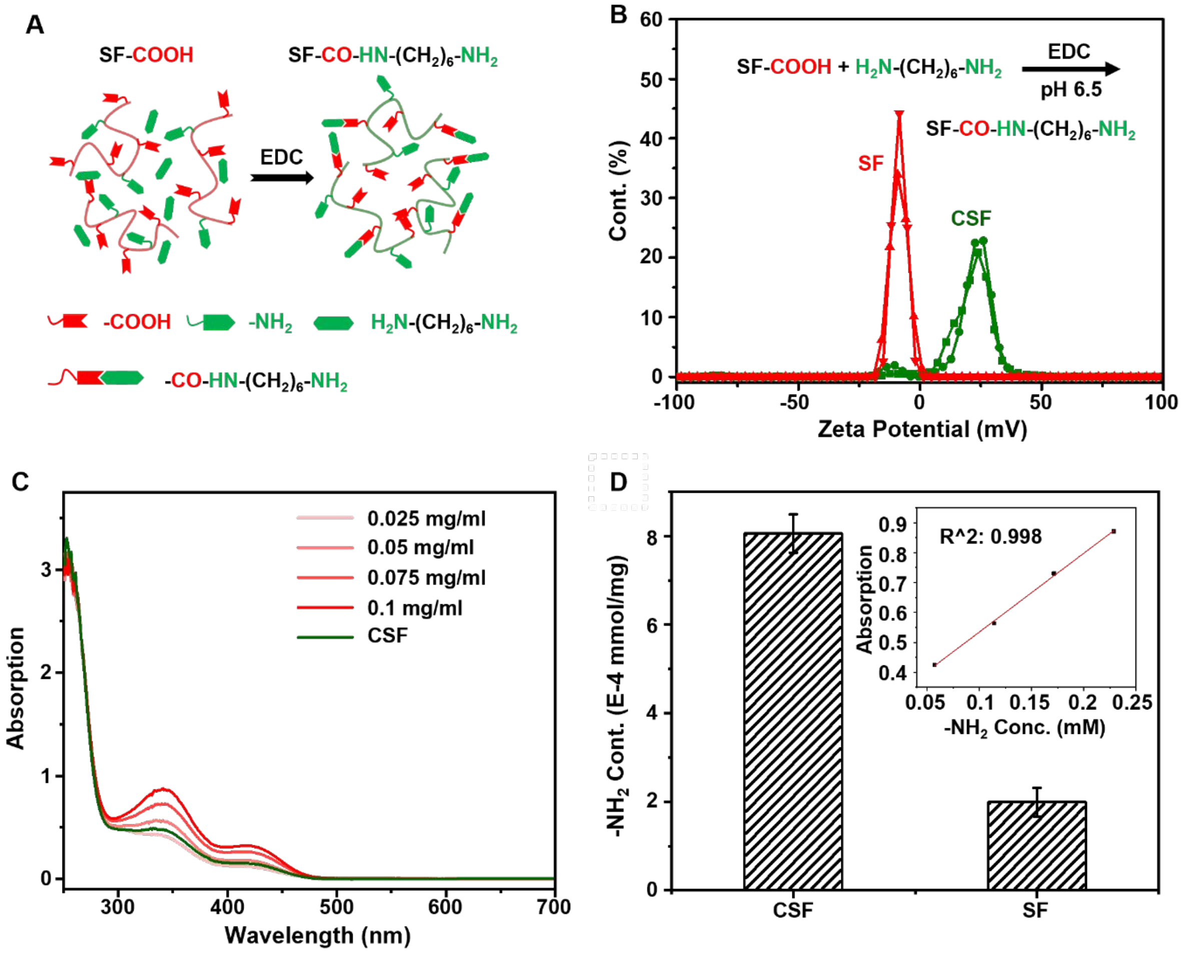

Supplementary Figure 1. Synthesis of cationized silk fibroin (CSF). (**A**) Scheme illustrating the preparation of CSF by an amination reaction under EDC activation. (**B**) Zeta potential of silk fibroin (SF) and CSF. (**C**) UV absorption spectra of CSF and BSA (standard curves determined at variable concentrations; see also (insert in (**D**)) after addition of 2,4,6-trinitrobenzene sulfonic acid (TNBSA) at 37 °C for 2 h to determine the primary amino content of CSF. (**D**) Amino contents of SF and CSF based on data shown in (**C**); inset shows calibration plot for BSA.

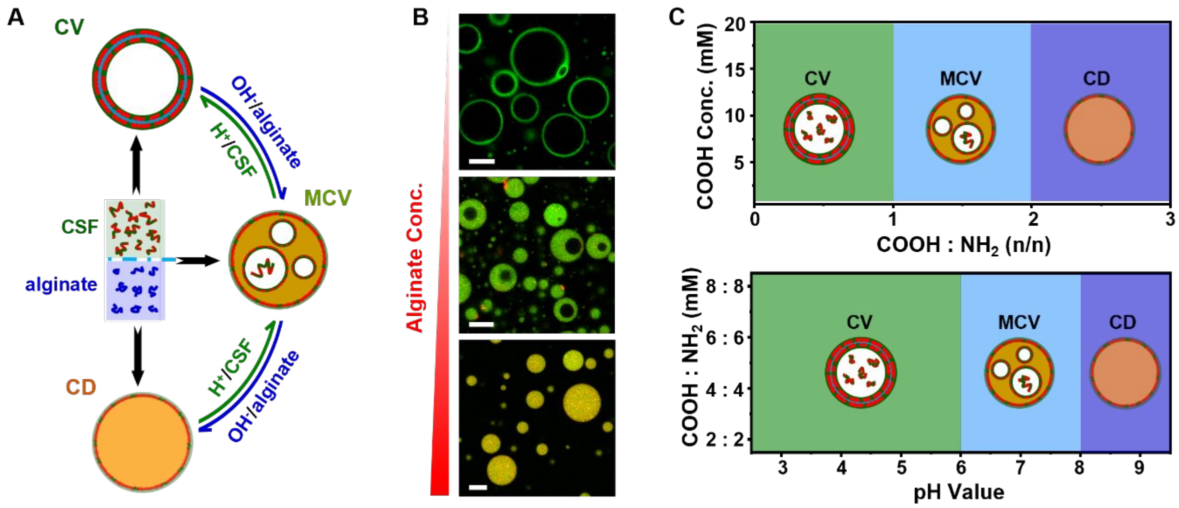

Supplementary Figure 2 Reconfiguration of alginate/CSF coacervates. (**A**) Scheme presenting the formation and reversible reconfiguration of complex alginate/CSF coacervate microstructures derived from changes in relative compositions ([COOH] (alginate) : [NH_2] (CSF)) or pH values; positively charged coacervate vesicles (**CV**), positively charged multi-compartmentalized coacervate vesicles (MCV) and close-to-neutral coacervate droplets (**C**). (**B**) LSCM images of alginate (red)/CSF (green) PCVs (top), MCVs (middle) and C**D**s (bottom) corresponding to increasing [COOH] (alginate) concentrations (4, 6 and 9 mM, respectively) at a constant [NH_2] (CSF) concentration of 4 mM. (**C**) Diagrams showing mapping of silk-based coacervate microstructures onto compositional [COOH] (alginate) : [NH_2] (CSF) ratios (top) and varying pH values (bottom). Scale bars are 20 µm.

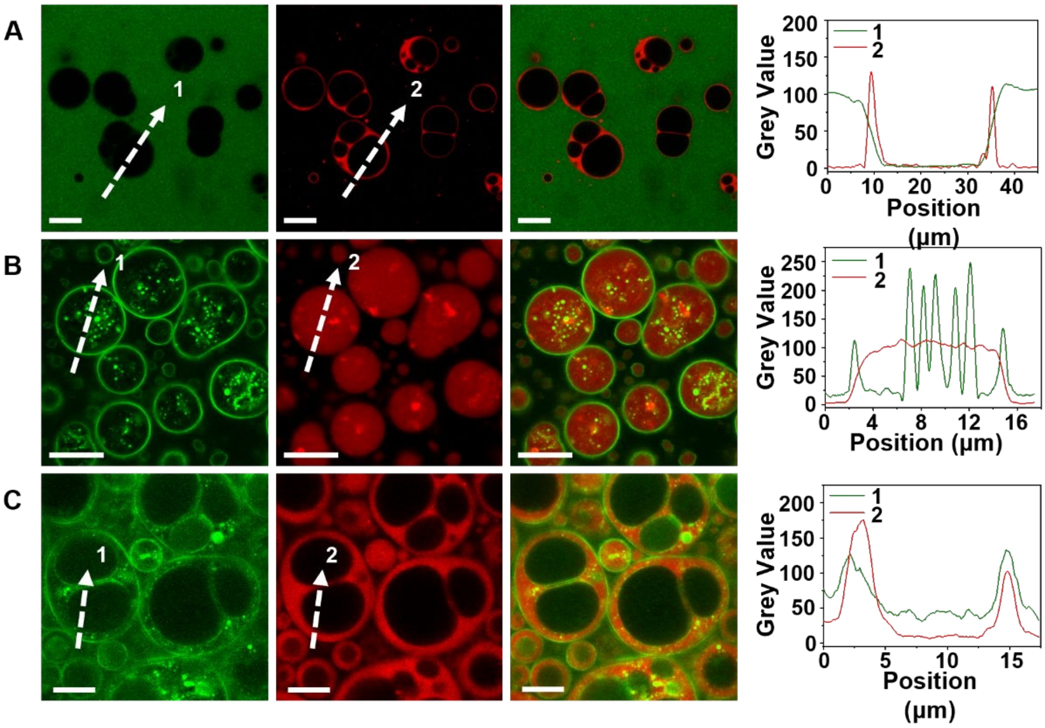

Supplementary Figure 3. Uptake studies of FITC-dextran into RITC-labelled alginate/CSF coacervate microstructures. (**A**) Fluorescence microscopy images of positively charged coacervate vesicles ([NH_2] 4 mM, [COOH] 3.9 mM, pH ~ 6.5) dispersed in aqueous FITC-Dextran (Mw: ~ 250 kD) showing absence of dextran penetration through the positively charged membrane. Arrows **1** and **2** correspond to line profiles for green and red fluorescence shown in the right column. (**B**) Coacervate droplets ([NH_2] 4 mM, [COOH] 8.0 mM) showing sequestration of dextran within the membrane-less microdroplets. Dextran undergoes segregative phase separation within the coacervate droplets and accumulates on the coacervate droplet surface. (**C**) Positively charged coacervate vesicles obtained by addition of CSF to sample (**B**) to decrease the ([COOH] (alginate) : [NH_2] (CSF)) charge ratio; the sequestered dextran in (**B**) remains preferentially concentrated within the compressed membrane-like coacervate phase of the positively charged coacervate vesicle. Negligible amounts of the polysaccharide are in the water lumen. Scale bars are 20 μm.

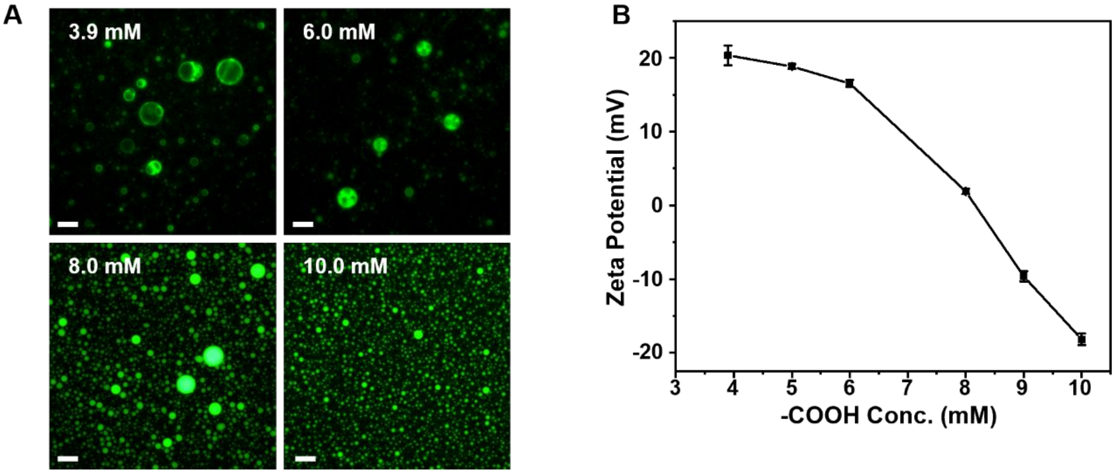

Supplementary Figure 4. Silk-based coacervate microstructures produced under increasing alginate [COOH] concentrations (3.9-10.0 mM) at constant [NH_2] = 4 mM). (**A**) LSCM images showing positively charged coacervate vesicles, positively charged multi-compartmentalized coacervate vesicles and large/small coacervate droplets at alginate concentrations of 3.9, 6.0, 8.0/10.0 mM, respectively. Scale bars are 20 µm. (**B**) Corresponding zeta potentials of the silk-based coacervate variants prepared at different [COOH] (alginate) concentrations (3.9-10 mM).

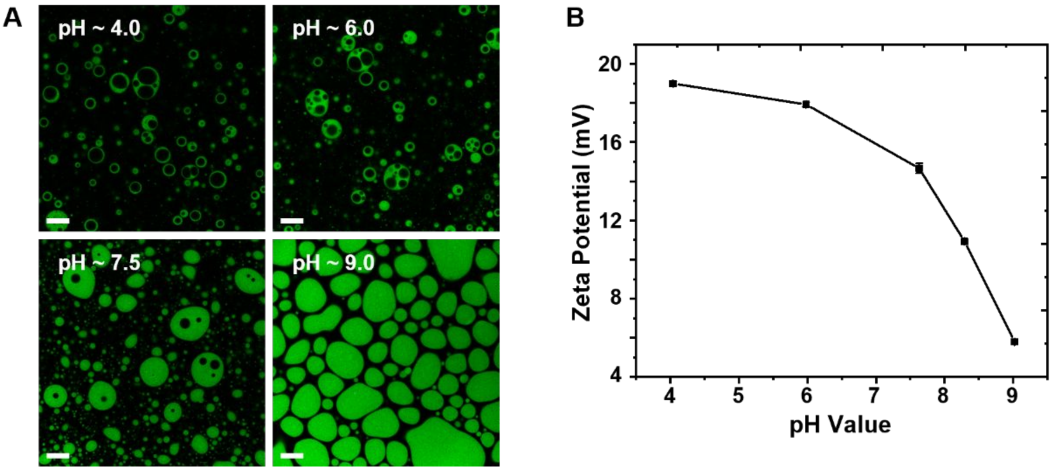

S**upplementary Figure 5.** Silk-based coacervate microstructures prepared at various pH values and constant CSF ([NH_2] = 4 mM) and alginate ([COOH] = 4 mM) concentrations. (**A**) LSCM images of positively charged coacervate vesicles, positively charged multi-compartmentalized coacervate vesicles with large/small water vacuoles, or coacervate droplets at approximate pH values of 4.0, 6.0/7.5 and 9.0, respectively; scale bars are 20 µm. (**B**) Corresponding zeta potentials of silk-based coacervate variants prepared at different pH values.

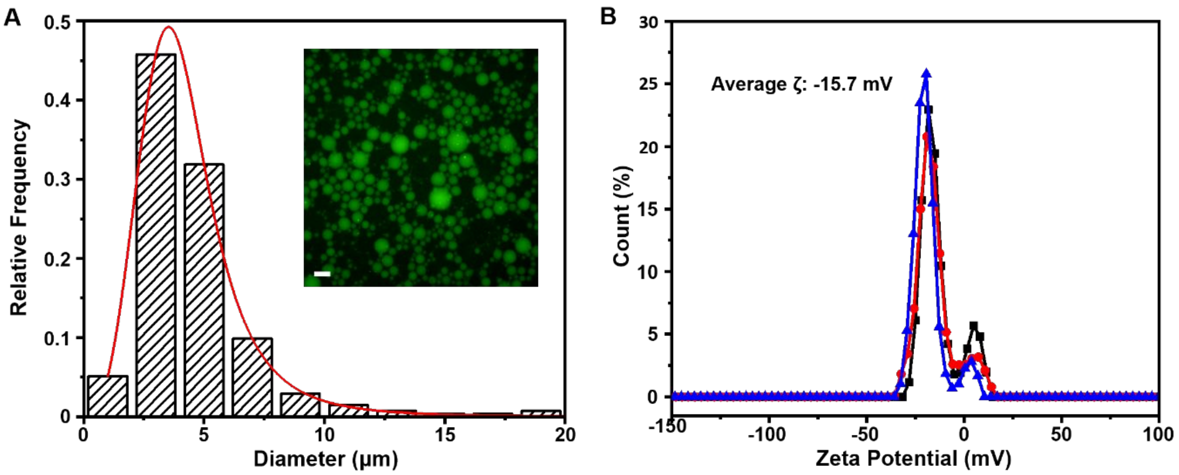

Supplementary Figure 6. Particle size distribution (**A**) and zeta potential profile (**B**) for proteinosomes used as guest protocells. Inset in (**A**) is a fluorescence microscopy image of an aqueous suspension of proteinosomes containing FITC-labelled gelatin (green fluorescence). Scale bar, 20 μm.

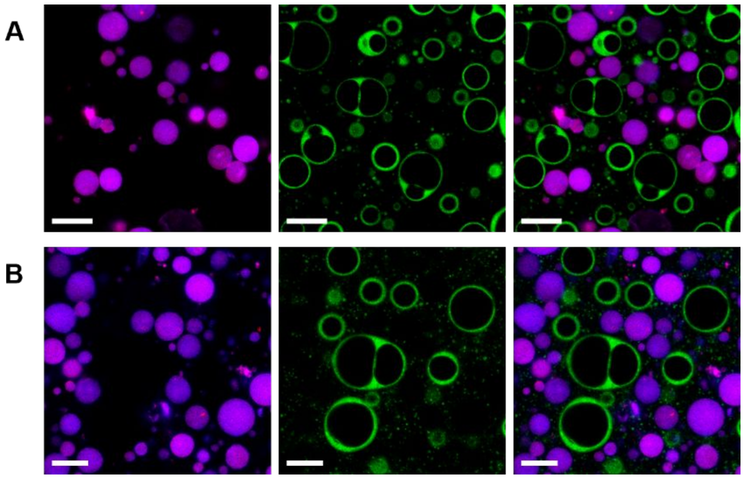

Supplementary Figure 7. Non-interacting mixed populations of Dylight 405-labelled proteinosomes containing RITC-labelled gelatin (purple fluorescence (blue + red)) and silk-based FITC-labelled positively charged coacervate vesicles (green fluorescence; [NH_2] 4 mM, [COOH] 4.0 mM, pH ~ 6.0). Left and middle columns are LSCM filtered images showing proteinosomes (purple) or FITC- positively charged coacervate vesicles (green), respectively. Right column shows corresponding merged images. Images were recorded at room temperature 0.5 h (**A**) and 24 h (**B**) after sample preparations. No fusion or wetting of the different populations is observed over 24 h. Scale bars are 20 μm.

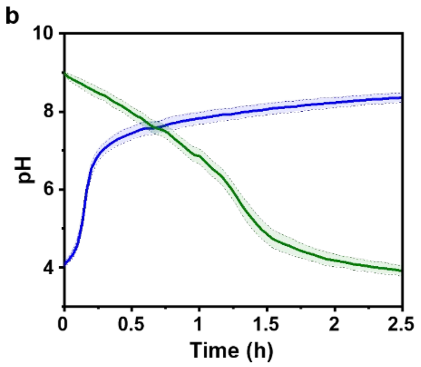

Supplementary Figure 8. Time-dependent plot of pH for a mixed population of GOx/urease-containing proteinosomes and positively charged coacervate vesicles ([NH_2]: 4 mM, [COOH]: 4.0 mM, initial pH_0 ~ 4.0) after addition of urea (40 mM) and formation of proteinosome-loaded coacervate droplets (blue plot). Corresponding plot after addition of glucose to the proteinosome-in-coacervate droplets (green, pH_0 ~ 9.0, 40 mM) leading to coacervate vesicles with encapsulated proteinosomes.

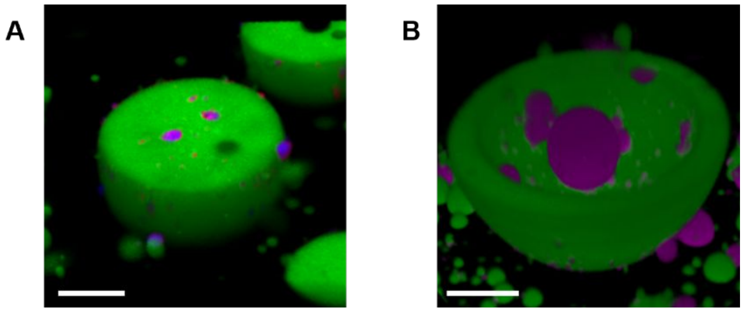

Supplementary Figure 9. 3D stacked LSCM images of a membrane-less proteinosome-in-coacervate droplet (**A**) and membranized proteinosome-in-coacervate vesicle (**B**). The proteinosomes appear in purple due to labelling with Dylight 405 (blue fluorescence) and presence of encapsulated RITC-labelled gelatin (red fluorescence). The coacervate vesicles are prepared with FITC-labelled CSF and alginate and exhibit green fluorescence. Scale bars are 20 µm.

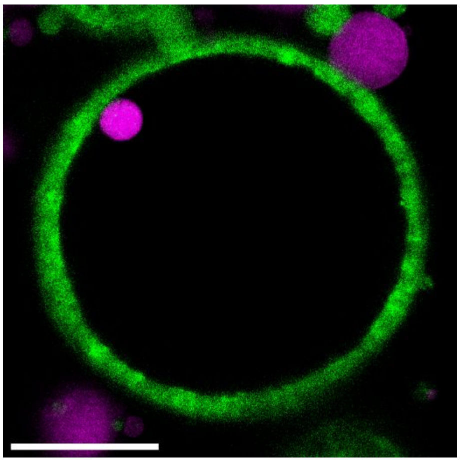

Supplementary Figure 10. LSCM image showing an individual proteinosome-in-coacervate vesicle at pH 4. Proteinosomes are labelled with Dylight 405 (blue fluorescence) and contain RITC-labelled gelatin (red fluorescence). The CSF/alginate coacervate phase is labelled using FITC-labelled CSF (green fluorescence). Scale bars, 20 µm.

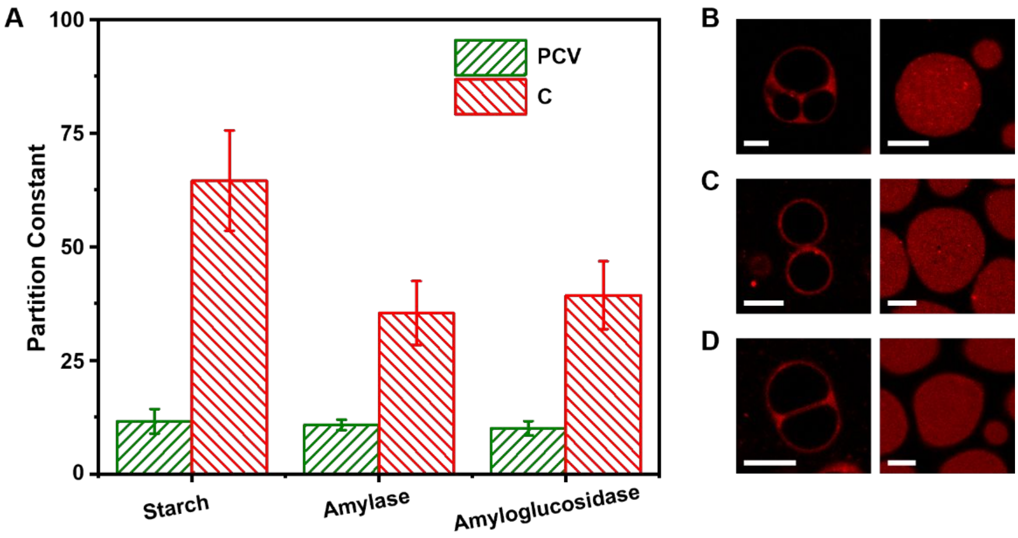

Supplementary Figure 11. Partition constants for RITC-labelled cargoes; soluble starch, amylase and amyloglucosidase (red fluorescence) in alginate/CSF coacervate droplets and vesicles. (**A**), plot showing partition constants of different cargoes; values were determined by calculating the ratio of red fluorescence intensity (grey value) inside and outside the coacervate droplets observed in LSCM images using Image J software. (**B-D**), LSCM images of positively charged coacervate vesicles (left) and membraneless coacervate droplets (right) with sequestered RITC-labelled starch (**B**), RITC-amylase (**C**) and RITC-amyloglucosidase (**D**). $[NH_2]$ (CSF), 4 mM; [COOH] (alginate), 4 mM; pH *ca*. 9.0. Scale bars, 20 µm.

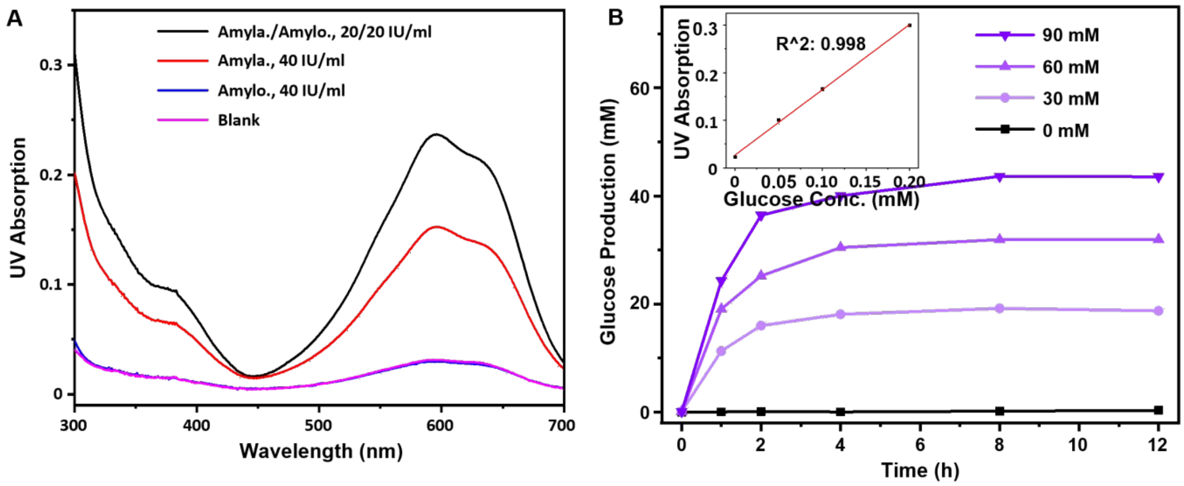

Supplementary Figure 12. Enzymatically driven starch hydrolysis. (**A**) UV spectra of hydrolysed starch after addition of amylase (Amyla.) or amyloglucosidase (Amylo.), or both (Amyla./Amylo). Spectra were recorded 3 h after addition of Amyla./Amylo. and show characteristic absorption peaks for soluble starch macromolecules. The spectrum obtained in the absence of the hydrolases is also shown (blank). (**B**) Time-dependent glucose production derived from the hydrolysis of soluble starch in the presence of Amyla./Amylo. (20/20 IU/ml) at total starch concentrations of 0, 30, 60 or 90 mM. Glucose concentrations were determined quantitatively using a GOx/HRP enzyme assay. UV absorption intensities of assay product 2,3 DAP were recorded after addition of *o*PD to the hydrolysed starch solution in the presence of GOx/HRP (1250/25 IU/ml). Inset is the standard curve of glucose concentration vs. UV absorption intensity at 490 nm.

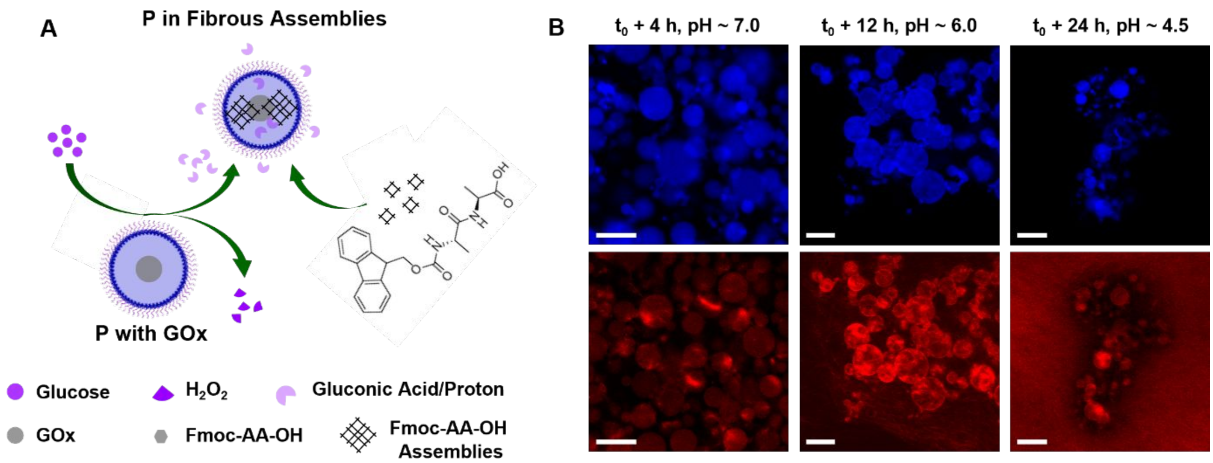

Supplementary Figure 13. (**A**) Scheme showing supramolecular assembly of Fmoc-AA-OH nano-filaments in semi-permeable GOx-containing proteinosomes exposed to glucose and the dipeptide. Addition of glucose and Fmoc-AA-OH results in acidification of the proteinosome interior, which in turn induces filament assembly initially inside and on the surface of the protocells, and then in the external environment leading to hydrogelation of the medium. (**B**) Time-series of fluorescence microscopy images showing populations of GOx-containing proteinosomes (blue; initial conditions, t_0, pH_0 ~ 9.0, $Na_2CO_3/NaHCO_3$ 10/10 mM) after addition of glucose (40 mM) and Fmoc-AA- OH (2 mM, 0.75 mg/ml). Self-assembled Fmoc-AA-OH filaments are observed as the pH decreases to 7, and extensively hydrogelled proteinosomes are observed at pH 6. Lower pH values give rise to filament formation in the external environment due to acidification of the bulk solution. Fmoc-AA-OH filaments are stained with Nile red. Scale bars are 20 µm.

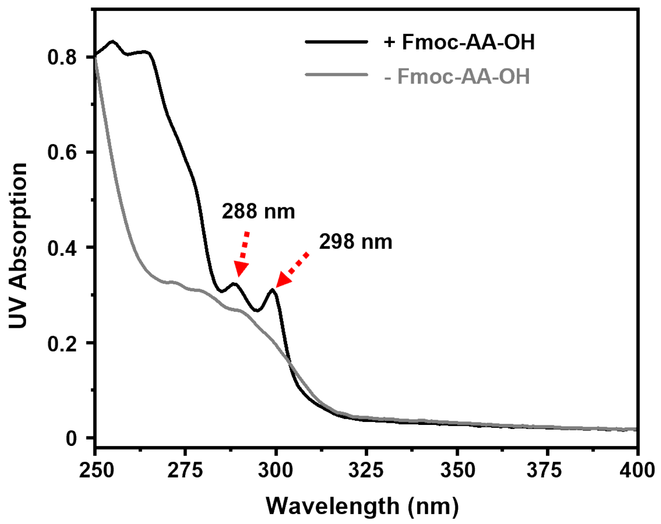

Supplementary Figure 14. UV spectrum (black plot) showing characteristic peaks for Fmoc-AA-OH in GOx/proteinosome-in-coacervate vesicles after supramolecular assembly ($[NH_2]$ 4 mM, [COOH] 4 mM, pH ~ 4.0, Fmoc-AA-OH 0.75 mg/ml). The modified guest/host protocells were collected by centrifugation (10,000 rpm, 5 min, 200 ml) and the supernatant removed, followed by treatment with 2 ml $Na_2CO_3/NaHCO_3$ buffer (100/100 mM) at pH ca. 9.0 to release Fmoc-AA-OH if present. The solutions were then analysed by UV spectroscopy. The spectrum associated with corresponding experiments undertaken without Fmoc-AA-OH is also shown (grey plot).

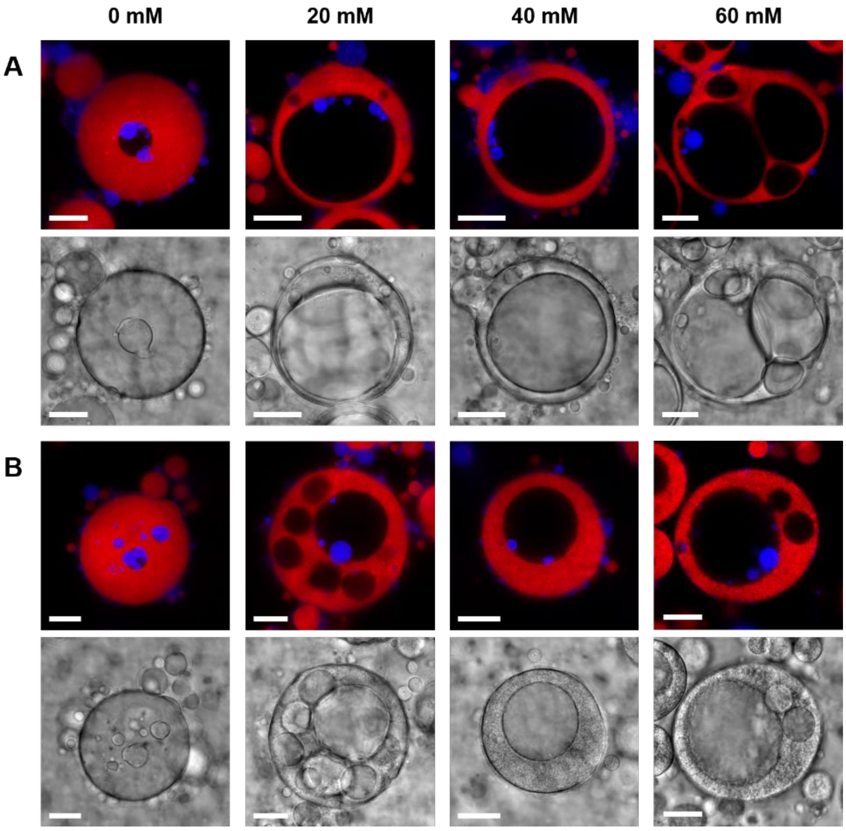

Supplementary Figure 15. LSCM (colour) and corresponding bright field (grey) images of individual GOx/proteinosome-in-coacervate vesicles (pH 4) prepared in the absence (**A**) or presence of Fmoc-AA-OH (0.75 mg/ml) (**B**). Glucose concentrations from left to right; 0, 20, 40 or 60 mM. Proteinosomes and alginate/CSF coacervate phase are labelled with Dylight 405 (blue fluorescence) or RITC-CSF (red fluorescence), respectively. Scale bars are 20 μm.

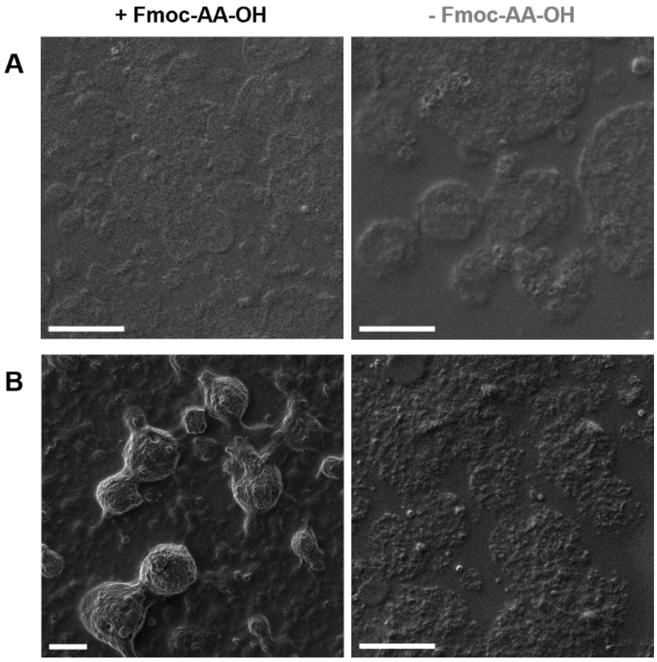

Supplementary Figure 16. SEM images of GOx/proteinosome-in-coacervate droplets (**A**, [NH_2] 4 mM, [COOH] 4.5 mM, pH ~ 9) and GOx/proteinosome-in-coacervate vesicles (**B**, pH 4, glucose 40 mM) prepared in the presence (left) or absence (right) of Fmoc-AA-OH (0.75 mg/ml). Scale bars, 20 µm. Samples were prepared by mounting a drop of the suspensions onto a PEG-functionalized cover slip followed by drying in a N_2 flow for 15 min, followed by Ag coating prior to SEM imaging. Evaporation-induced collapse of the all the samples except proteinosome-in-coacervate vesicles with Fmoc-AA-OH nanofilaments is observed, indicating the increased mechanical robustness of these hybrid protocells.

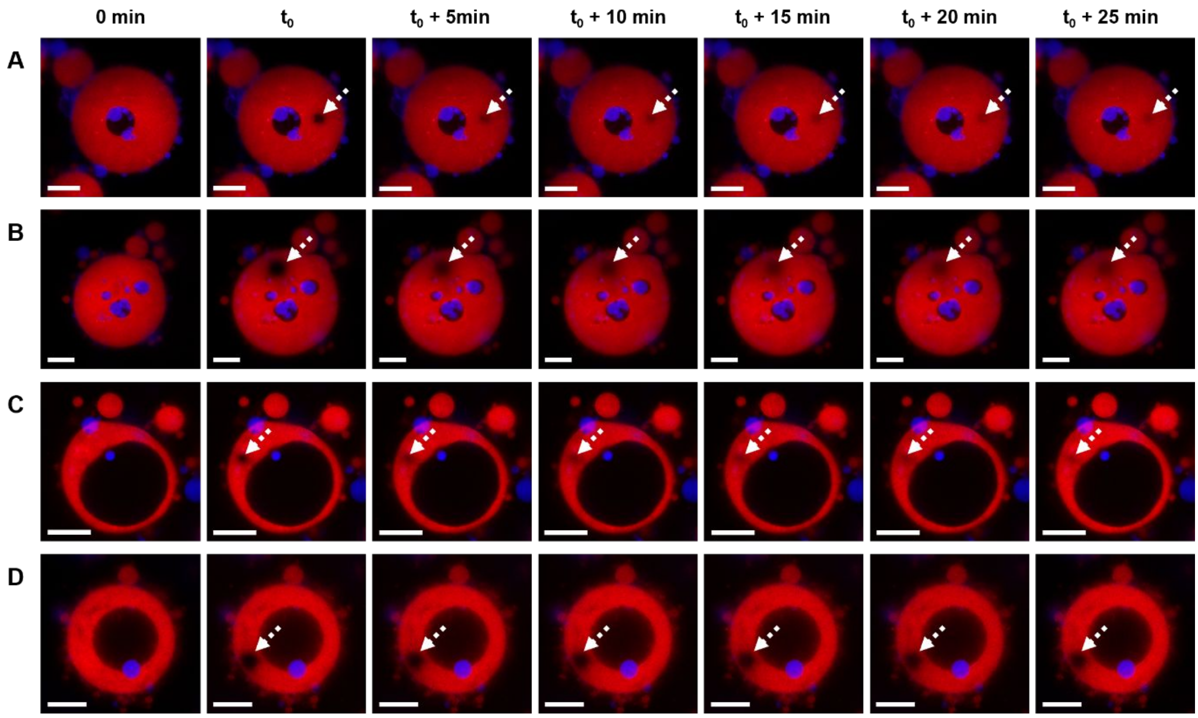

Supplementary Figure 17. FRAP data for individual GOx/proteinosome-in-coacervate droplets prepared at pH 9 without (**A**) or with Fmoc-AA-OH (**B**, 0.75 mg/ml) and GOx/proteinosome-in-coacervate vesicles prepared at pH 4 by adding glucose (20 mM) without (**C**) or with Fmoc-AA-OH (D, 0.75 mg/ml). White arrows highlight the photo-bleached areas in the coacervate phase. Proteinosomes and alginate/CSF coacervate phase are labelled with Dylight 405 (blue fluorescence) or RITC-CSF (red fluorescence), respectively. Scale bars are 20 µm.

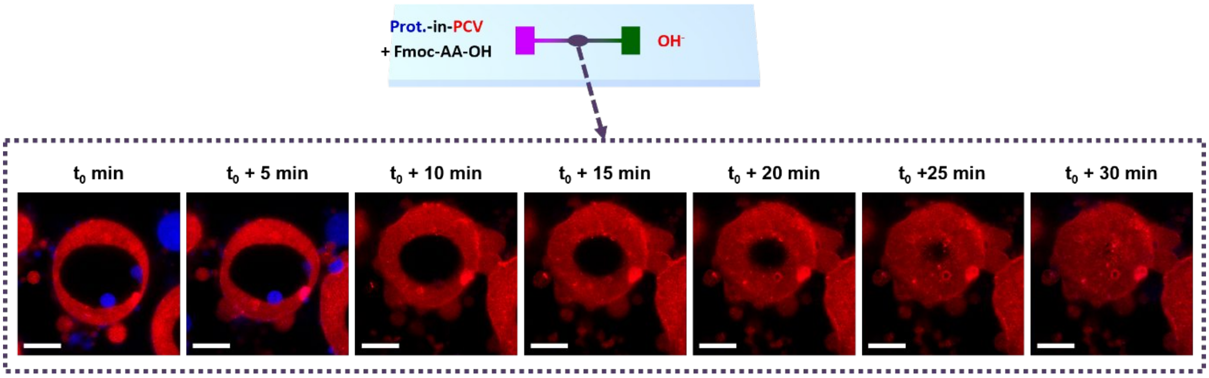

Supplementary Figure 18. Time-series of fluorescence microscopy images showing transition of an individual Fmoc-AA-OH-reinforced GOx/proteinosome-in-coacervate vesicle (t_0, pH 4) to a proteinosome-containing coacervate droplet as the pH increases in a gradient of NaOH (0.005 M). Disassembly of Fmoc-AA-OH nanofilaments in the coacervate membrane domain has no effect on reconfiguration to the proteinosome-in-coacervate droplet. The captured proteinosomes (blue) are not observed in the images recorded after 10 min due to their movement out of the focal plane. Scale bars are 20 µm.

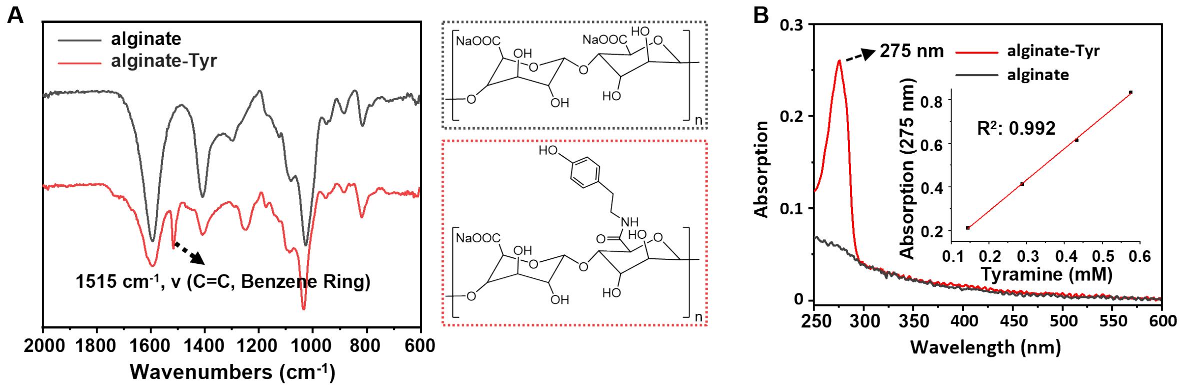

Supplementary Figure 19. Characterization of alginate-Tyr. (**A**) FTIR spectra of alginate (black) and alginate-Tyr (red). (**B**) Absorption profiles of alginate (black) and alginate-Tyr (red). The absorption peak at 275 nm confirms the presence of the tyramine group. Inset shows calibration curved used to determine the Tyr content in alginate-Tyr (ca. 15 % (n/n)). Polysaccharide concentration, ca. 0.3 mg/ml.

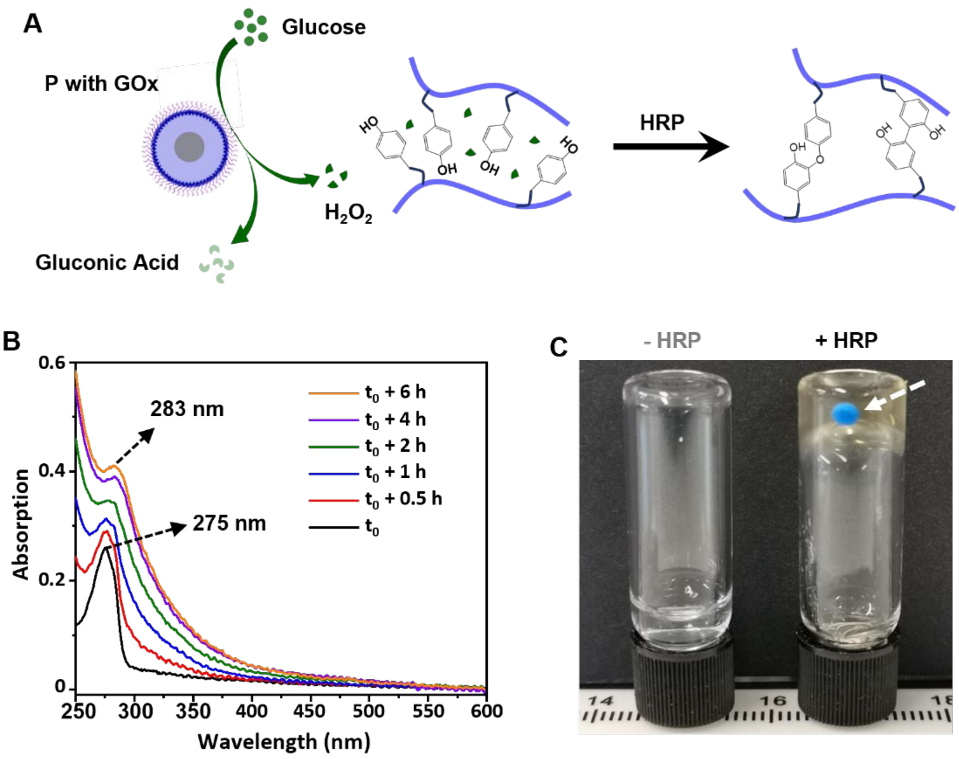

Supplementary Figure 20. Enzymatically triggered crosslinking of alginate-Tyr by GOx-containing proteinosomes in the presence of HRP. (**A**) Scheme showing input of glucose, activation of proteinosome-encapsulated GOx, co-production of gluconic acid and H_2O_2, and subsequent dimerization of tyramine side groups by HRP-mediated oxidation in the presence of H_2O_2 to produce a chemically crosslinked hydrogel. (**B**) Time-dependent UV/VIS absorption profiles of an alginate-Tyr solution (0.25 mg/ml) containing glucose (30 mM), GOx-containing proteinosome (GOx, 3 mg/ml, 1 x 10^7 proteinosomes per ml) and HRP (7.5 IU/ml). A red shift from 275 to 283 nm occurs due to dimerization of the tyramine groups. (**C**) Image of inverted alginate-Tyr solutions (5 mg/ml) containing glucose (30 mM) and GOx-containing proteinosomes in the absence (left) or presence (right) of HRP. Immobilization of a stirring bar (blue) confirms extensive hydrogelation in samples containing HRP.

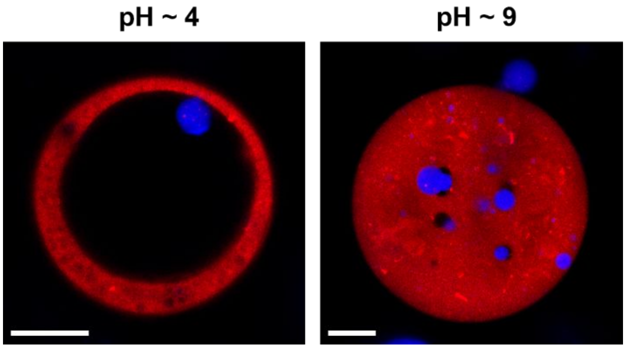

Supplementary Figure S21. LSCM images of alginate-Tyr/CSF coacervate vesicles (pH 4 (left)) and coacervate droplets (pH 9 (right)). Scale bars, 20 µm.

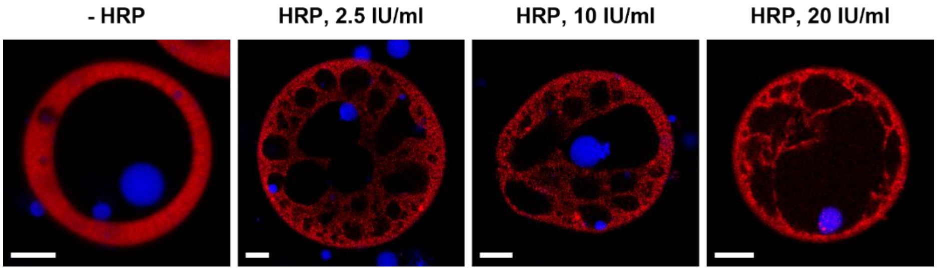

Supplementary Figure S22. LSCM images of alginate-Tyr/CSF (red) coacervate vesicles containing GOx/proteinosomes (blue). Images are recorded after addition of glucose (30 mM) in the absence of HRP (left) or presence of HRP (2.5-20 IU/ml) to initiate crosslinking. Samples were incubated at room temperature for 24 h. Concentrations; [NH_2] (RITC-CSF) 4 mM; [COOH] (alginate-Tyr) 5 mM. Scale bars, 20 µm.

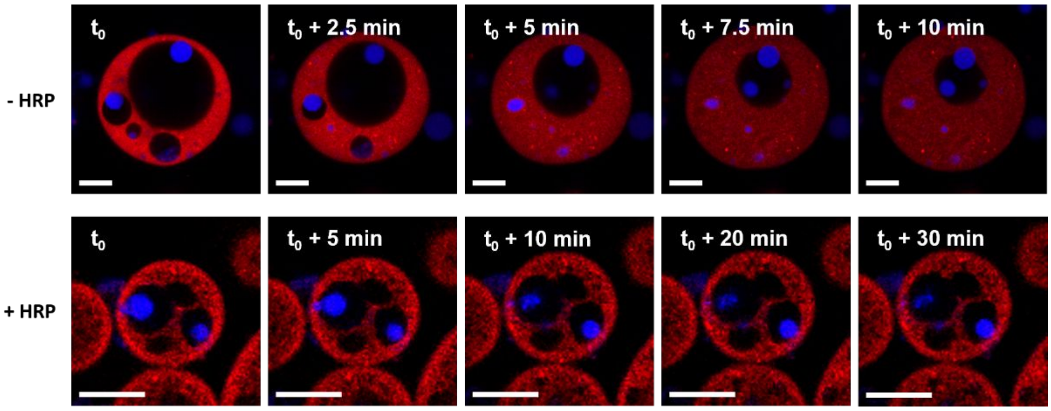

Supplementary Figure S23. Time-dependent LSCM images of non-crosslinked (-HRP, top row) or crosslinked (+HRP, bottom row) alginate-Tyr/CSF (red) coacervate vesicles containing GOx/proteinosomes (blue) and exposed to a NaOH concentration gradient (0.025 M). Crosslinking prevents reconfiguration into coacervate droplets. Scale bars 20 µm.

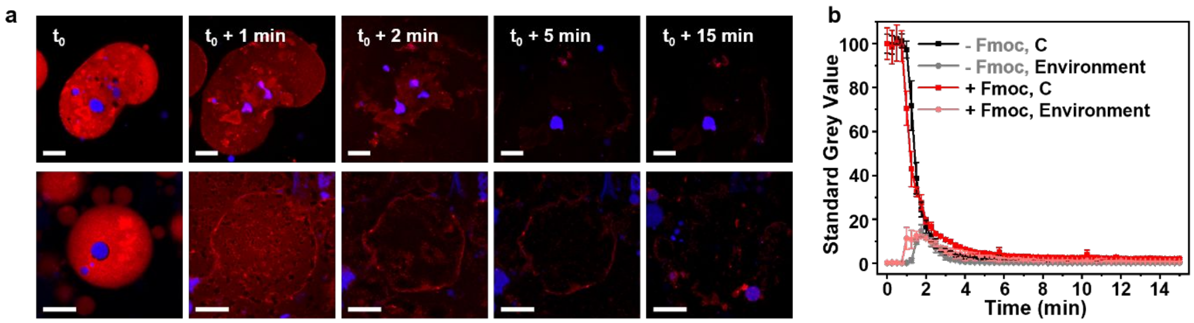

Supplementary Figure 24. (**A**) Time-series of LSCM images showing salt-induced disassembly of a single proteinosome-in-coacervate droplet without (top row) or with (bottom row) sequestered Fmoc-AA-OH at pH 9 and in the presence of NaCl (4 M). In both cases, intact proteinosomes are released into the external environment. Proteinosomes are labelled with Dylight 405 (blue fluorescence); alginate/CSF coacervate phase is labelled with RITC-CSF (red fluorescence). Scale bars, 20 µm. (**B**) Time-dependent changes in red fluorescence (RITC-CSF) for proteinosome-in-coacervate droplets shown in (**a**). Grey values were measured in the centre of the hybrid protocell and in the external environment.

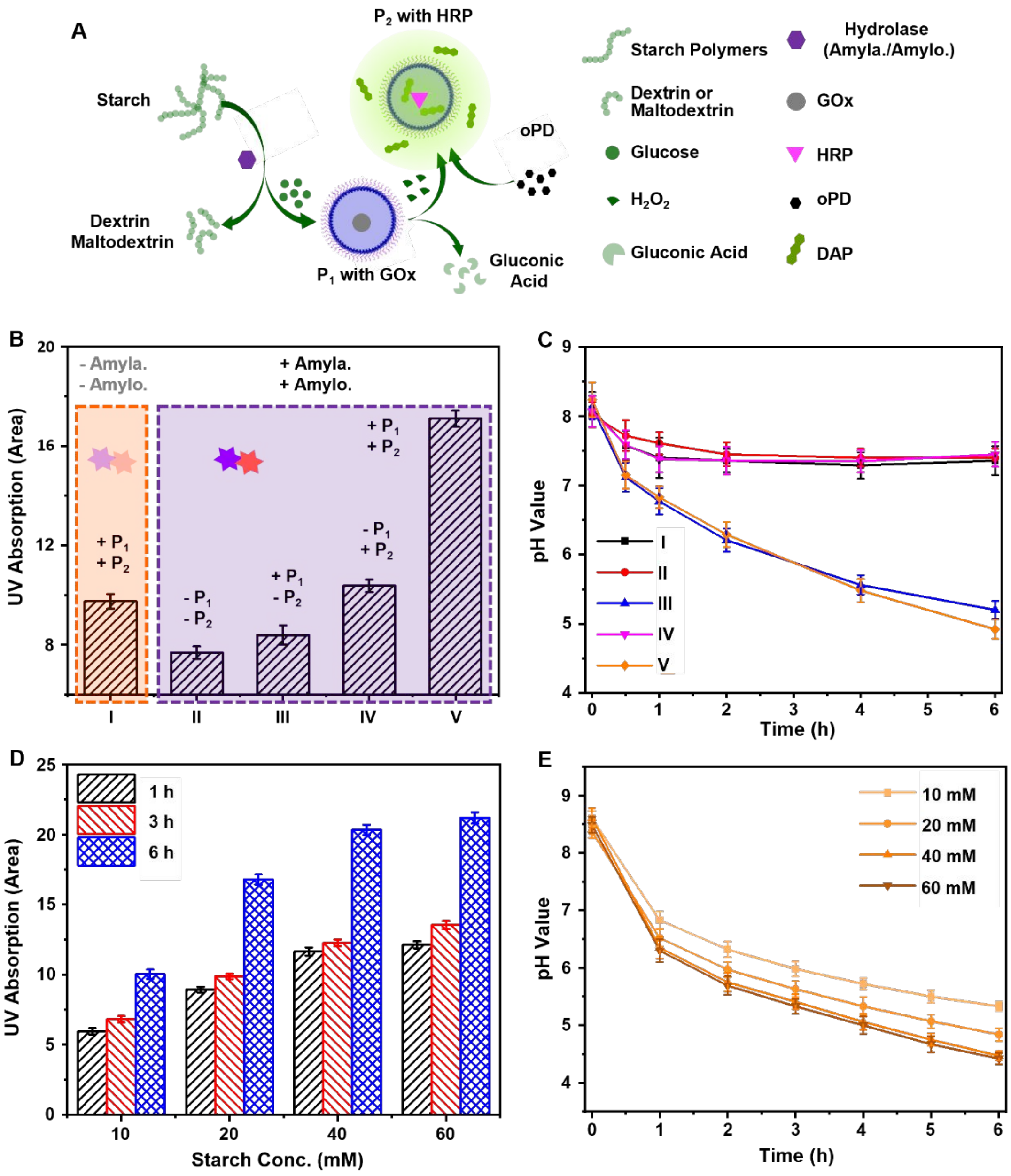

Supplementary Figure 25. Control experiments undertaken in binary populations of GOx (grey dots)- or HRP (pink triangles)-containing proteinosomes in the absence of alginate/CSF coacervate droplets demonstrate enzyme-mediated starch hydrolysis, signalling and fluorescence output. (**A**) Scheme showing starch hydrolysis in the bulk solution followed by production of glucose and initiation of the GOx/HRP cascade between the two proteinosome populations leading to 2,3-DAP production and a green fluorescence output. (**B**) Graph showing UV absorbance values associated with 2,3-DAP production in presence (II-V) or absence (I) of amylase (Amyl)/amyloglucosidase (Amylo) for a binary population GOx-containing proteinosomes (P_1) and HRP-containing proteinosomes (P_2) (V), single populations of P_1 or P_2 (III, IV), or without proteinosomes (II). The absorbance was determined by calculating the peak area of the UV absorption (360-600 nm). (**C**) Time-dependent changes in pH values associated with samples I-V in (**B**). (**D,E**) Time-dependent changes in UV absorption with different starch concentrations (**D**) and associated changes in pH (**E**) for sample (V).

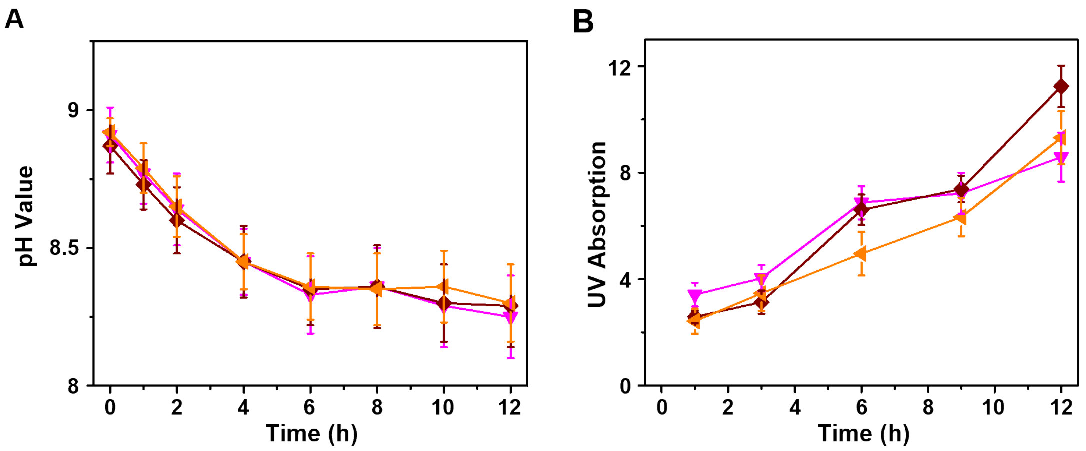

Supplementary Figure 26. Plots showing time-dependent changes in pH (**A**) and absorbance at 495 nm (**B**) in **CV** containing hydrolase and starch (pink triangles) and **P_1/P_2@CV** without hydrolase (dark red diamonds) or starch (orange triangles).

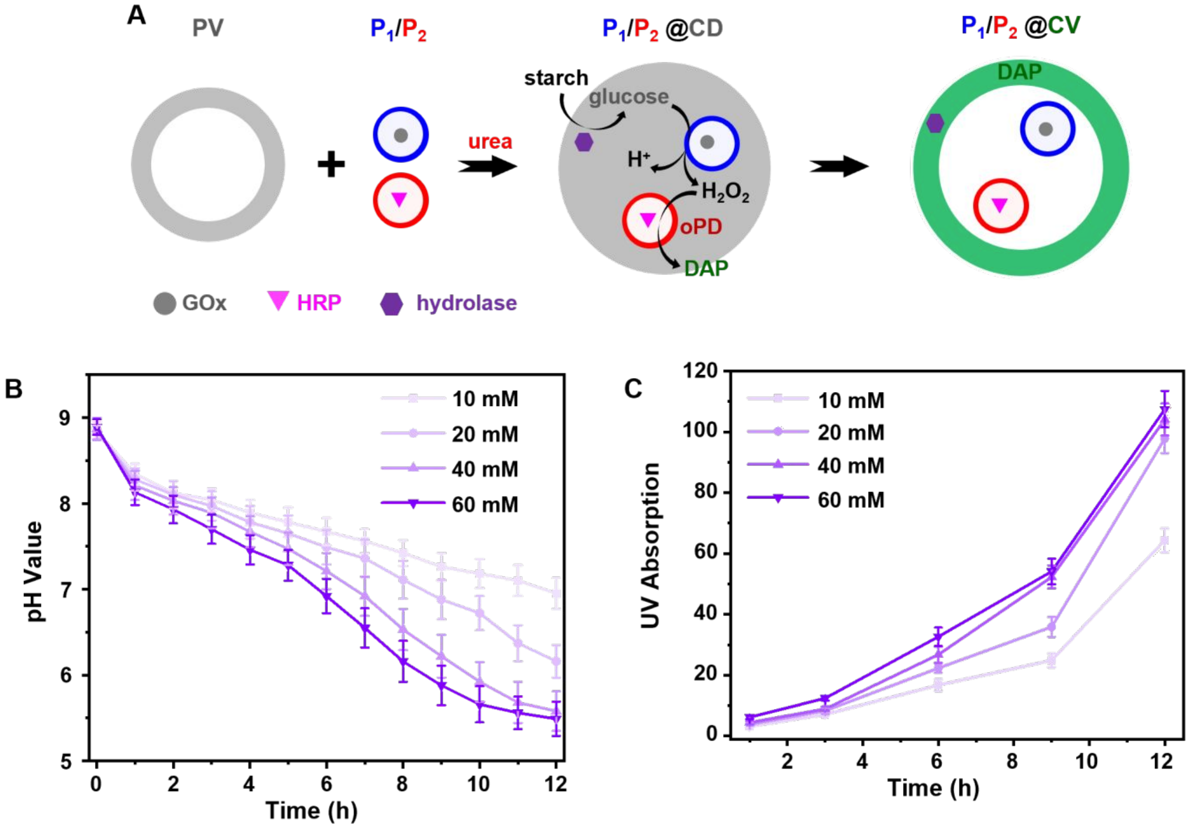

Supplementary Figure 27. (**A**) Scheme showing fuel-driven three-enzyme network via a hybrid protocell reconfiguration pathway. (**B**) and (**C**) are the consequent changes in pH values and UV absorption of proteinosome-in-coacervate droplets/vesicles suspensions ($[NH_2]$ 4 mM, [COOH] 4.0 mM, pH_0: ~ 9.0) in the presence of starch (10, 20, 40, 60 mM).

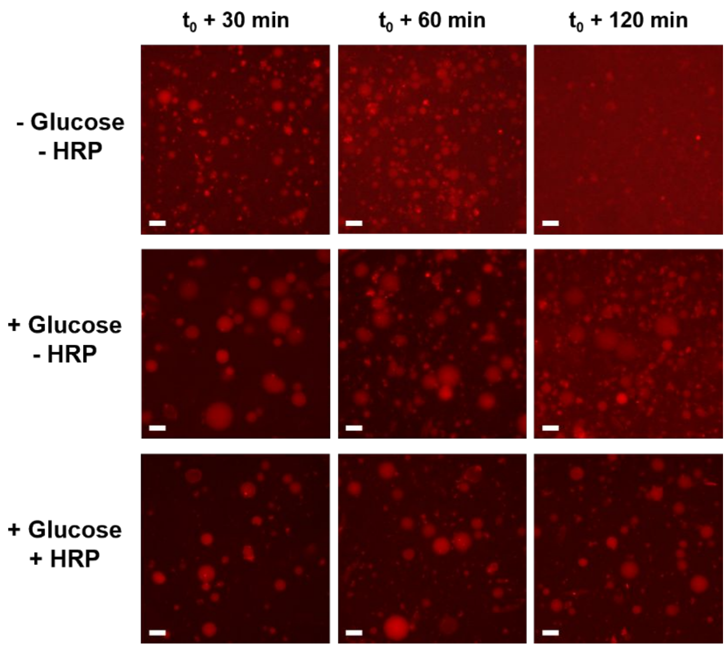

Supplementary Figure 28. Control experiment: time-sequence of fluorescence images of disulfide-crosslinked proteinosomes containing GOx (4 mg/ml) and RITC-gelatin (6 mg/ml) undergoing TCEP (5 mM) disassembly without glucose and HRP (-glucose/-HRP) or with glucose and HRP at respective concentrations of 20 mM/0 IU/ml (+ glucose/-HRP), and 20 mM + 15 IU/ml (+glucose/+HRP). TCEP was added 30 min after glucose addition. More intact proteinosomes are observed in the presence of both glucose and HRP. Scale bars are 20 µm.

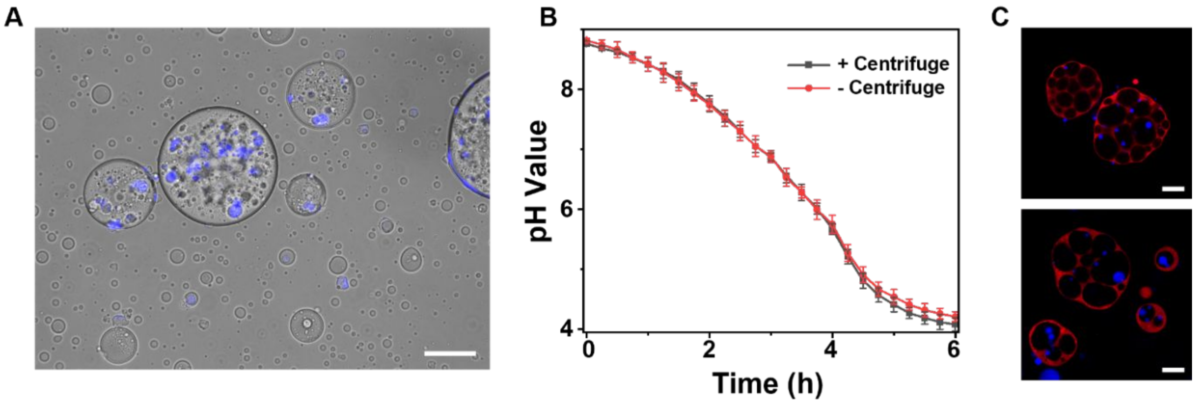

Supplementary Figure 29. (A) Merged optical and fluorescence microscopy images of proteinosome-containing coacervate droplets (Psome@CD) with a proteinosome number density of 3.3 x 10^6/ml, equivalent to the number density of the captured population (30%) determined at a proteinosome : coacervate vesicle number ratio of 1 : 2 (see Figure 1 of the manuscript). Over 90 % of the proteinosomes (blue fluorescence) are transferred into the membrane-less coacervate droplets by mechanical transfer under centrifugation (5000 rpm, 5 min) at pH 9.0. (B) Time-dependent changes in pH of a centrifuged or non-centrifuged Psome@CD suspension showing identical pH profiles (glucose concentration, 40 mM), indicating that a captured proteinosome number density of 3.3 x 10^6/ml is sufficient to induce the transition of proteinosome-containing coacervate droplets (pH 9) to proteinosome-containing coacervate vesicles (pH 4). However, the pH decrease occurs at a reduced rate, requiring 4 h to reach a pH of 4.0 compared with 1.5 h in the experiments shown in Figure S8. (C) LSCM images of proteinosome-in-coacervate vesicles (Psome@CVs) produced by glucose addition to Psome@CD prepared without (upper image) or with (bottom image) centrifugation. Scale bars, 50 µm (A) and 20 µm (C).

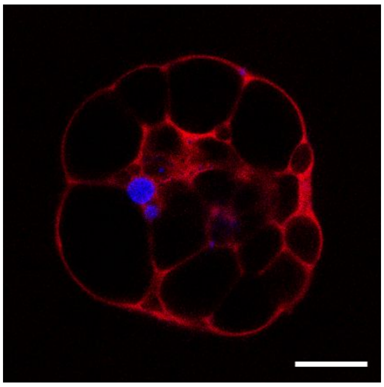

Supplementary Figure 30. LSCM image showing a multi-compartmentalized coacervate vesicle (red fluorescence) containing proteinosomes (blue fluorescence) primarily associated with the coacervate phase after addition of glucose. GOx and urease are sequestered within the alginate/CSF coacervate and the proteinosomes are non-enzymatic.

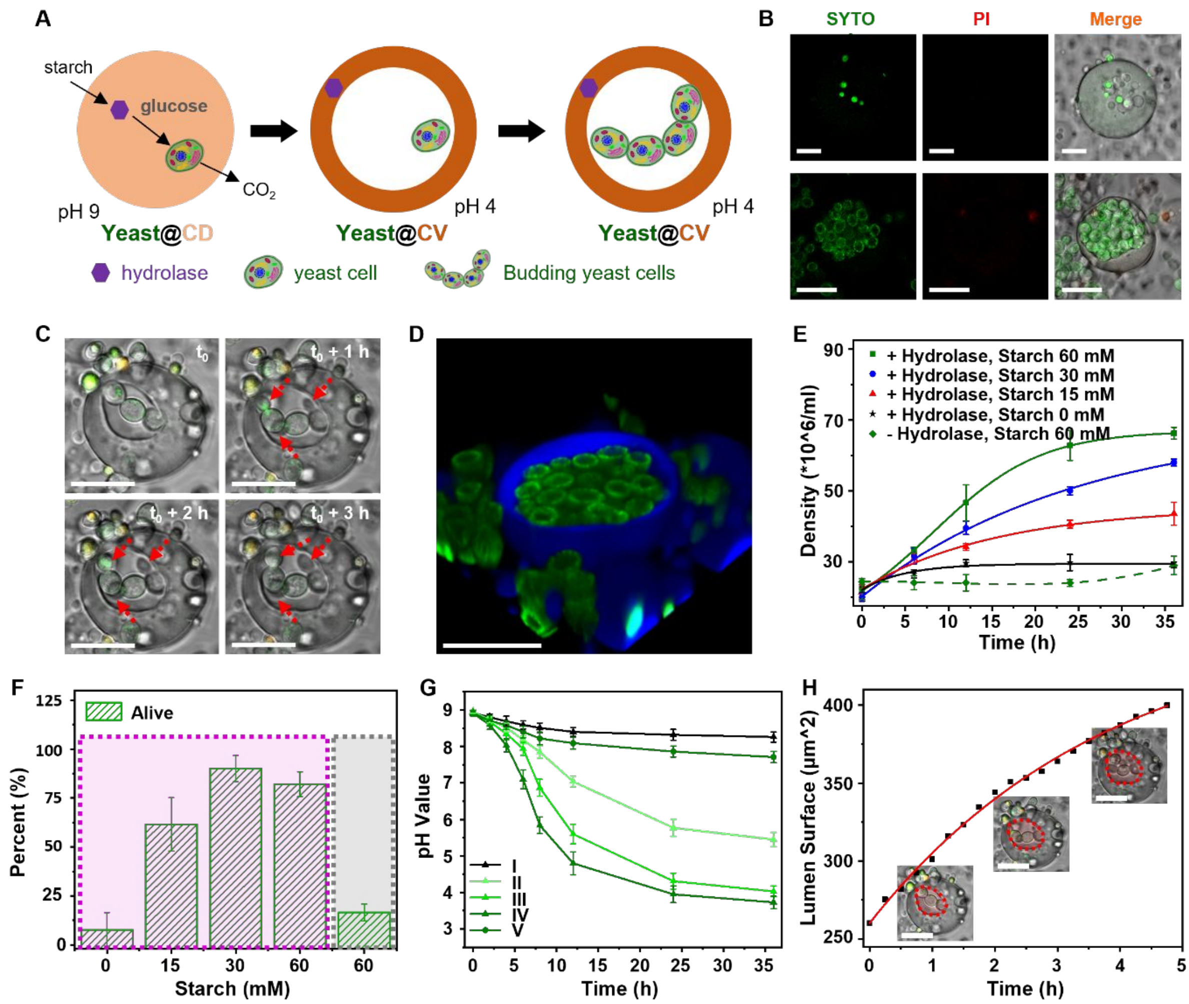

Supplementary Figure 31. Self-driven capture of yeast cells in reconfigurable coacervate vesicles. (**A**) Live yeast cells (ca. 2.5 x 10^7/ml) are initially incorporated at pH 9.0 into membrane-less alginate/CSF coacervate droplets (**CD**, light orange circle, [NH_2] 4 mM, [COOH] 4.5 mM) containing sequestered starch hydrolases (purple hexagons, amylase/amyloglucosidase). Addition of soluble starch induces endogenous production of glucose which subsequently initiates fermentation by the captured yeast cells to produce CO_2 and acidification (pH 4) of the protocell environment, resulting in membranization/reconfiguration of the coacervate droplets into coacervate vesicles (**CV**, dark orange circle) with captured living yeast cells. Compression of the coacervate phase during osmotically induced expansion results in expulsion of the yeast cells into the aqueous-filled lumen of **CV** where they proliferate by budding division to produce a densely packed viable population. (**B**) LSCM images of individual **yeast@CD** (upper row) and **yeast/PCV** (bottom row); the latter were recorded 36 h after starch (60 mM) addition. Yeast cells were stained with SYTO 9 (green, live cells) and PI (red, dead cells). (**C**) Time-sequence of images showing budding yeast division (red arrows) inside a single **CD** that is undergoing reconfiguration into **CV**. (**D**) 3D-stacked image of a single **yeast@CV** with densely packed living yeast cells in the lumen. (**E**) Plots showing increasing density of yeast cells over time in a **yeast@CD/PCV** suspension at different starch concentrations (0-60 mM) in the presence of starch hydrolases. Cell growth increases at higher starch concentrations. Minimal cell division is observed in the absence of the starch hydrolases. (**F**) Bar graphs showing percentage of yeast cells alive 36 h after starch addition to a **yeast@C** suspension in the presence (purple background) or absence (grey background) of starch hydrolases. Live/dead staining of the encapsulated yeast cells indicated that over 75% of the cells remained viable at starch concentrations higher than 30 mM. (**G**) Plots of time-

dependent changes in pH for **yeast@CD/CV** suspensions prepared at different concentrations of starch and starch hydrolases. Groups (I)-(IV) contain both starch (0, 15, 30, 60 mM) and starch hydrolase (40/20 IU/ml). Group (V) contains starch (60 mM) but no starch hydrolase. (**H**) Plot showing time-dependent increase in lumen volume during the reconfiguration of **yeast@CD** into **yeast@/CV** due to yeast-induced acidification of the protocell environment. Scale bars are 20 µm.
